# APPEAL‐2: A pan‐European qualitative study to explore the burden of peanut‐allergic children, teenagers and their caregivers

**DOI:** 10.1111/cea.13719

**Published:** 2020-09-15

**Authors:** Audrey DunnGalvin, Katy Gallop, Sarah Acaster, Frans Timmermans, Lynne Regent, Sabine Schnadt, Marcia Podestà, Angel Sánchez, Robert Ryan, Pascale Couratier, Mary Feeney, Betina Hjorth, Helen R. Fisher, Katharina Blumchen, Andrea Vereda, Montserrat Fernández‐Rivas

**Affiliations:** ^1^ University College Cork Cork Ireland; ^2^ Acaster Lloyd Consulting London UK; ^3^ Nederlands Anafylaxis Netwerk Dordrecht The Netherlands; ^4^ The Anaphylaxis Campaign Farnborough UK; ^5^ Deutscher Allergie‐ und Asthmabund Mönchengladbach Germany; ^6^ Food Allergy Italia Padua Italy; ^7^ Asociación Española de Personas con Alergia a Alimentos y Látex Madrid Spain; ^8^ Aimmune Therapeutics London UK; ^9^ Association Française pour la Prévention des Allergies Paris France; ^10^ St Thomas' Hospital London UK; ^11^ Astma‐Allergi Danmark Roskilde Denmark; ^12^ King's College London London UK; ^13^ Division of Pneumology, Allergology and Cystic Fibrosis Department of Children and Adolescent Medicine University Hospital Frankfurt Frankfurt am Main Germany; ^14^ Hospital Clínico San Carlos IdISSC Madrid Spain

**Keywords:** burden, conceptual models, food allergy, paediatrics, peanut allergy, quality of life

## Abstract

**Background:**

Allergy to Peanuts ImPacting Emotions And Life (APPEAL‐1) was a recent European multi‐country questionnaire survey that highlighted the negative impacts of peanut allergy (PA) on quality of life. A follow‐on qualitative study, APPEAL‐2, further assessed the burden of PA and associated coping strategies through semi‐structured interviews.

**Objective:**

To gain qualitative insight on the strategies used to cope with and manage PA and the impact of these strategies on the quality of life of children, teenagers and caregivers.

**Methods:**

This cross‐sectional qualitative study was conducted in eight European countries: the United Kingdom, France, Germany, Ireland, Spain, Italy, Denmark and the Netherlands. Semi‐structured interviews were conducted with children (aged 8‐12 years) and teenagers (aged 13‐17 years) with self‐/proxy‐reported moderate or severe PA and with parents/caregivers of children or teenagers (aged 4‐17 years) with moderate or severe PA. Data were analysed using thematic analysis; data saturation was assessed. Two conceptual models were developed to illustrate the impacts of PA and coping strategies used to manage them for (a) individuals with PA and (b) parents/caregivers of children with PA.

**Results:**

107 participants were interviewed: 24 children, 39 teenagers and 44 caregivers. The conceptual models illustrated themes related to coping and control, driven by the fear of PA reactions, and the associated emotional, social, relationship and work impacts. Factors moderating these impacts included social attitudes and support, child‐caregiver relationship and coping strategies used.

**Conclusions and Clinical Relevance:**

The APPEAL‐2 results substantiate the findings of APPEAL‐1; the results also suggest that the severity of experience with PA may not correlate with perception of its overall burden and show variable impacts by country.

## 1 INTRODUCTION

Management of peanut allergy (PA) centres on strict avoidance of peanuts and emergency treatment in the case of accidental exposure leading to a severe reaction or anaphylaxis. The constant vigilance required can have a profoundly negative impact on the health‐related quality of life of individuals with PA and their caregivers.^1^, ^2^, ^3^, ^4^ Children with PA report poorer health‐related quality of life, greater fear of adverse events, and more anxiety about eating than children with insulin‐dependent diabetes^1^ and have poorer quality of life than children with seafood allergy.^5^ Compared with their siblings, children with PA have significantly poorer health‐related quality of life in physical, psychological and social dimensions, both at school and in general.^2^

Allergy to Peanuts ImPacting Emotions And Life (APPEAL) is a two‐part study conducted across eight European countries to evaluate the psychosocial burden of living with PA. APPEAL‐1 utilized a quantitative, cross‐sectional online survey to investigate the experience of adults and children with PA and caregivers, including demographic and clinical factors and the impact of PA on psychosocial parameters and health‐related quality of life. APPEAL‐1 found that individuals experience frustration, stress, uncertainty and low levels of confidence in managing their PA.^6^, ^7^

The analysis of quantitative questionnaires is limited to data provided by answers to predetermined questions. This approach may generate a bias towards the preconceptions of the questionnaire's creators and fail to adequately capture the views, experiences and impacts that are important to those living with PA. A qualitative approach that elicits information on subjects’ everyday lives may provide data that more accurately reflect the complexity of real‐life situations and thus reveal unmet healthcare needs and suggest innovative ways to resolve them.^8^

A few qualitative studies to date have focused specifically on individuals with PA and their families.^9^, ^10^ Although conceptual models have been developed for food allergy in general,^11^, ^12^ no previously published studies have proposed conceptual models that illustrate the complex impacts of PA on the lives of those affected. Research assessing the impacts of PA specifically is needed because PA is lifelong in the majority of patients, and more frequently than most other food allergies,^13^ and is associated with comparatively high rates of severe reactions, anaphylaxis and fatal anaphylaxis in Western countries.^14^, ^15^, ^16^, ^17^ Furthermore, previous studies have not looked specifically at the real‐world impact of PA‐related coping strategies on patients and their families. Finally, no cross‐sectional research has explored the broader health‐related quality of life impact of PA across different age groups and European countries.

APPEAL‐2 was a large qualitative study designed to further explore key areas of impact identified in APPEAL‐1 and to ensure that all concepts important to those impacted by PA were captured. In contrast to APPEAL‐1, which collected only proxy‐reported data from caregivers regarding the impact of PA on children and teenagers, APPEAL‐2 includes self‐reported data from children and teenagers, as well as proxy report from caregivers. The objectives were to investigate the health‐related quality of life burden of living with PA on children, teenagers and caregivers across Europe, the coping strategies used to cope with this burden, and to develop conceptual models that provide a holistic overview of the data collected.

## 2 METHODS

### 2.1 Study design

APPEAL‐2 employed a cross‐sectional study design using qualitative methods to explore the impact of PA on children and teenagers with PA and on parents/caregivers (“caregivers”) of individuals with PA. Semi‐structured interview guides (see Appendix S1), developed with clinical experts and patient advocacy groups across all participating countries, allowed participants to spontaneously describe how PA affected them. Interviewers employed pre‐specified probes if concepts were not raised. Caregivers were asked about the impact of PA on their child and on their own life. All interviews began with the question, “First, I would like to ask you some questions to give us a general picture of your experience with peanut allergy,” and almost all subsequent questions reminded the participants that their responses should related specifically to PA (eg, “…because of your peanut allergy”). The study was approved by the Western Independent Review Board (IRB tracking/approval number 20182422).

The study was conducted in the United Kingdom, France, Germany, Spain, Italy, the Netherlands, Denmark and Ireland. Specialist recruitment panels engaged participants drafted from databases of individuals willing to participate in research studies. Eligible participants were aged 8‐17 years, separated in categories of children aged 8‐12 years (included only in the United Kingdom, France and Germany) and teenagers aged 13‐17 years (included in all countries) with a diagnosis of moderate or severe PA (self‐/proxy‐rated) or caregivers of children and teenagers (aged 4‐17 years) with a diagnosis of moderate or severe PA (self‐/proxy‐rated). All children and teenagers had experienced at least one accidental reaction to peanut. Recruitment aimed for minimums of 50% of the sample in each country with severe PA and 25% (of the total population) who reported having used an adrenaline autoinjector (AAI) or experienced a life‐threatening event.

### 2.2 Data collection procedures

All participants gave informed consent (or assent: children/teenagers) prior to participation. Interviewers conducted 30‐ to 60‐minute interviews in the local language, following the semi‐structured interview guide, between October 2018 and January 2019. Demographic and clinical information was also gathered (see Appendix S1). In the United Kingdom, France and Germany, all child and teenager interviews and some caregiver interviews were conducted in person. In other countries, all interviews were conducted by telephone. To allow participants to speak freely and openly, they were interviewed alone. Each interview was recorded and transcribed and, if applicable, translated into English. Caregivers who participated (proxy‐ and self‐report) and caregivers of children who participated were remunerated for their time in the study.

### 2.3 Analysis

Qualitative analysis of the transcripts was conducted using thematic analysis.^18^ A team of analysts coded the qualitative text of the transcripts using a coding frame. Analysis was assisted by MAXQDA, a qualitative software tool. Saturation, the point at which no new information is obtained from additional qualitative data,^19^ was assessed using saturation tables.^20^ Based on consensus of all analysts, those who coded the transcripts using the concepts identified during the analysis developed the conceptual model. Core concepts, or themes, included in the conceptual models included moderators, coping and control techniques, and impacts, which were comprised of multiple factors. Core concepts and themes, and the multiple factors comprising them, were identified by their frequency of mention in the interview transcripts and consensus of the transcript analysts.

## 3 RESULTS

There were 107 participants across the three groups and eight countries. Demographic and clinical information is summarized in Table 1. A high proportion of the overall sample had other allergies, with approximately one‐third of each cohort allergic to *tree* nuts in addition to peanuts. One‐third of the overall sample had no other food allergies. Three‐quarters of each cohort had an AAI prescription for their PA. Among all participants, 57% had severe PA (self‐ or caregiver‐reported), 50% had used an AAI, and 40% had experienced a life‐threatening event.

**TABLE 1:** Population demographics and clinical characteristics

		Children (n = 24)^a^	Teenagers (n = 39)^b^	Caregivers (n = 44)^c^
Country	UK	8	8	8
	France	8	8	8
	Germany	8	8	8
	Spain	0	3	4
	Ireland	0	3	4
	Italy	0	3	4
	Netherlands	0	3	4
	Denmark	0	3	4
Age, years	Mean (SD)	9.3 (1.4)	15.4 (1.4)	39.4 (6.2)
	Range	8‐12	13‐17	25‐49
				Child's age
	Mean (SD)			9.2 (3.5)
	Range			4‐17
Sex, female	n (%)	6 (25)	26 (67)	40 (91)
				Child's sex
	% Female			39
Age at first reaction, years	Mean (SD)	3.5 (2.1)	7.3 (4.1)	3.9 (3.5)
Age at diagnosis, years	Mean (SD)	4.0 (2.2)	6.8 (4.6)	4.0 (3.1)
Other food allergies^d^	n (%)	14 (58)	31 (79)	26 (59)
Tree nut allergy	n (%)	8 (33)	13 (33)	17 (39)
PA present in other family members	n (%)	3 (13)	11 (28)	7 (16)
AAI prescribed	n (%)	18 (75)	29 (74)	33 (75)

Abbreviations: AAI, adrenaline autoinjector; PA, peanut allergy; SD, standard deviation; UK, United Kingdom.

^a^ Fourteen caregivers of child participants were also interviewed.

^b^ Three caregivers of teenage participants were also interviewed.

^c^ Thirty caregivers of teenage and 14 of child participants.

^d^ Celery, cow milk and dairy products, egg (hen's), fish, fruit, meat or poultry, mustard, peach, seeds (eg poppy), sunflower, sesame, shellfish/crustacean/molluscs, soya beans/other legumes, sulphites and wheat/gluten.

### 3.1 Qualitative analysis results

**FIGURE 1:**
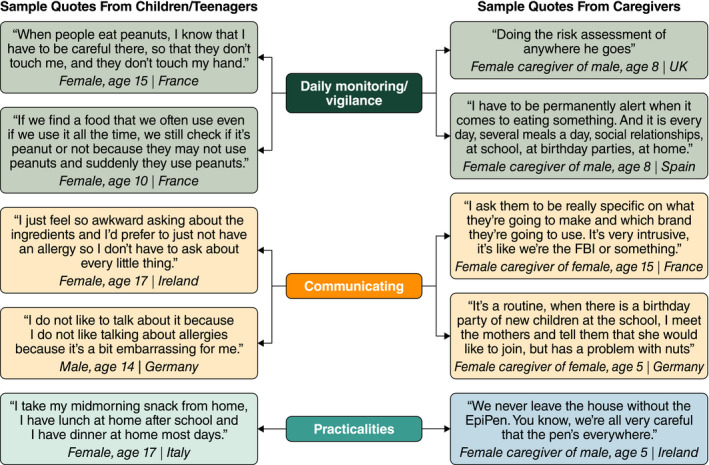
Example quotations from children, teenagers and caregivers illustrating the data relating to coping and control

**FIGURE 2:**
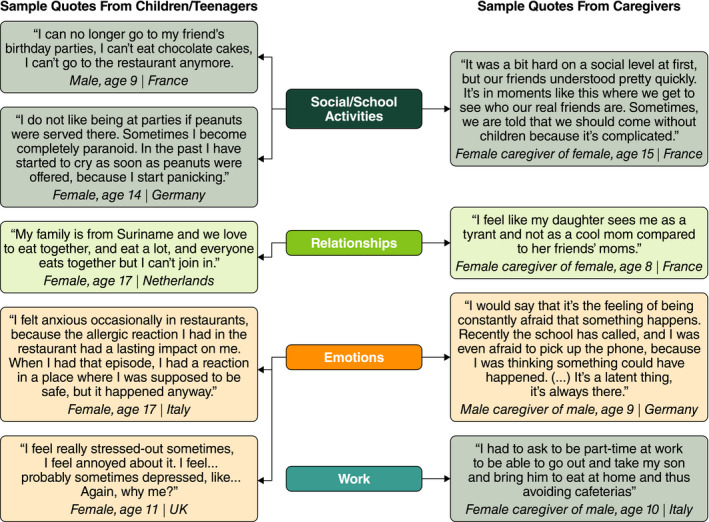
Example quotations from children, teenagers and caregivers illustrating the data relating to impacts on HRQL. HRQL, health‐related quality of life

Data saturation was reached when no new codes were added in the last 17 teenager interviews, the last three child interviews and the last six caregiver interviews. All core concepts or themes were reported spontaneously across all groups. Sample quotations illustrating how these core concepts were expressed are shown in Figures 1 and 2.

#### 3.1.1 Coping and control

Coping and control behaviours used to avoid accidental exposures to peanut could be categorized into three themes: daily monitoring/vigilance/avoidance, communicating, and practicalities and planning.

#### 3.1.2 Daily monitoring/vigilance/avoidance

All participants reported using some level of monitoring and avoidance to ensure safety from peanut exposure. For children and teenagers, this included checking ingredients, being aware of what others were eating and staying away from people eating peanuts, not sharing food with friends, and hygiene practices such as frequent handwashing or asking others to wash their own hands. Many teenagers and children reported bringing their own food to school and social events or always eating at home. Caregivers described needing to be alert and vigilant and to constantly “risk‐assess” situations for the possibility of peanut exposure and their child's safety.

#### 3.1.3 Communicating

All caregivers and teenagers and over half of children reported having to communicate about their PA with others in their environment, such as restaurant staff. Some teenagers felt self‐conscious, awkward or embarrassed when disclosing their PA, which was among the most difficult aspects of living with PA. Almost a third of children and a small number of teenagers did not want others to know about their PA and actively chose not to disclose it, some because of embarrassment, others to avoid teasing or bullying. Communication for caregivers included needing to meet, call or text message other adults and food providers before social events to enquire about the food being offered and its preparation.

#### 3.1.4 Practicalities and planning

Most children and teenagers reported carrying emergency medication (AAIs and antihistamines) with them and keeping spares in other locations; some reported carrying medications only if food was involved and/or they expected to eat. Caregivers reported having to ensure they or their child always carried the medication and that additional AAIs or antihistamines were at school or family members’ homes. For caregivers, buying and preparing food were major, time‐consuming aspects of managing their child's PA. Travel required packing the emergency kit, checking expiration dates on medications, communicating about food options and preparing food. Caregivers often mentioned needing to determine suitable places to eat and the distance to a hospital or pharmacy beforehand.

**FIGURE 3:**
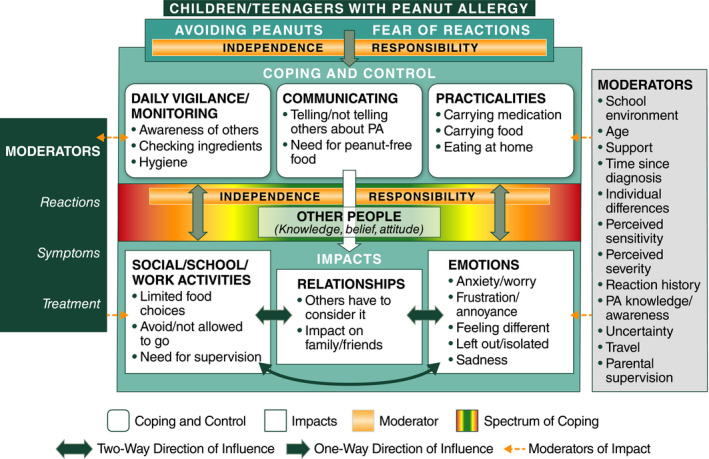
Conceptual model of the impact of PA on children and teenagers. The colour spectrum between “Coping and Control” and “Impacts” represents the range of reported behaviours. Red indicates either a highly vigilant or a careless approach, both of which can have a negative impact. Green indicates a more balanced approach and a positive impact. PA, peanut allergy

**FIGURE 4:**
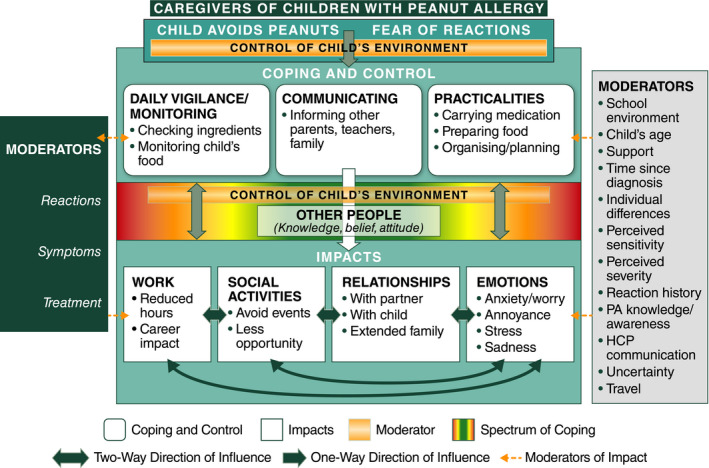
Conceptual model of the impact of PA on caregivers. The colour spectrum between “Coping and Control” and “Impacts” represents the range of reported behaviours. Red indicates either a highly vigilant or a careless approach, both of which can have a negative impact. Green indicates a more balanced approach and a positive impact. HCP, healthcare professional; PA, peanut allergy

Over half of teenagers, some children and many caregivers described elements of coping and control as the most difficult aspect of living with PA, although to varying degrees within the sample. Some strategies helped mitigate the likelihood of accidental exposure with limited impact on quality of life, while others appeared to trade control for quality of life (Figures 3 and 4).

#### 3.1.5 Impact of coping strategies used to manage peanut allergy

The negative impacts of avoiding peanuts and the coping and control behaviours were many and varied. The main areas of impact of living with PA were social and school activities, relationships, emotional impacts and work (caregivers only).

##### Social and school activities

Almost all teenagers and children and many caregivers reported a negative impact of PA on their social activities. For teenagers and children, using “avoidance” as a strategy included not only restaurants but avoidance of certain places (eg cinemas) and missing activities with friends. Some parents did not allow their children to attend social events (eg younger children could go only if a parent attended), causing children to “miss out” on many social activities. Almost a quarter of caregivers preferred to avoid social events if peanuts were served or if they would have no control over the environment. Occasionally, caregivers were asked not to bring their child to an event, or attended events without their child. Participants discussed not eating or having limited food choices at parties; some brought their own food with them. With regard to using communication as a strategy, most teenagers reported negative experiences when going to restaurants with friends, including embarrassment at having to declare their PA or being treated unkindly by staff. The need for strategic planning conferred a strong burden with caregivers often having to contact hosts or caterers to ask about food options and make them aware or remind them of their child's PA before participating in any social activities.

##### Relationships

Most participants reported that coping and control strategies for PA affected their relationships in some way. Negative effects on family relationships included arguments arising from forgetting an AAI, increased supervision vs siblings and repercussions for sibling relationships. Reported positive effects included stronger parent–child bonds due to the PA, although a small proportion of parents reported that their children perceived them as too controlling. Approximately half of children and teenagers and a small number of caregivers reported negative impacts on friendships. Children and teenagers felt left out or envious due to being unable to attend social events and share food with others. Caregivers reported needing to trust and burden friends to accommodate their child's PA. Several participants reported incidents of teasing or bullying relating to their PA. A quarter of teenagers reported an impact of PA on dating and on relationships with boyfriends/girlfriends. Approximately one in four caregivers said their child's PA had a negative impact on their relationship with their partner.

##### Emotional impact

Emotional impacts of coping with PA affected almost all participants. The most common emotional impacts included anxiety, worry and/or fear, often related to experiencing an allergic reaction or anticipating the risk of one. Some participants discussed feelings of worry or uncertainty related to interpreting food labels that stated “may contain traces of peanuts” (the word “trace” is not scientifically or legally defined) as individuals were unsure whether the food was safe to eat. Caregiver anxiety was rooted in a lack of control; approximately half reported worrying about having less control of their child's food and environment as the child became more independent. Several teenagers and some caregivers also reported being fearful of having to use their AAI, feeling nervous if they forgot it and worrying that it might not work. Children also reported fear or worry related to new environments or contact with someone who had eaten peanuts. Other common emotional impacts included feelings of frustration or annoyance, sadness or disappointment, stress, embarrassment and feeling different from others.

##### Work (caregivers)

Approximately a quarter of caregivers reported that they had reduced their working hours outside the home or decided to work part‐time to enable their child to eat lunch at home because of managing their child's PA. A similar proportion of caregivers also reported needing to take time off work to supervise their child on school trips or other activities, or to attend appointments. PA was not reported to impact job performance; however, some caregivers felt that reducing their working hours hampered career progression/job opportunities.

### 3.2 Country similarities and differences

**FIGURE 5:**
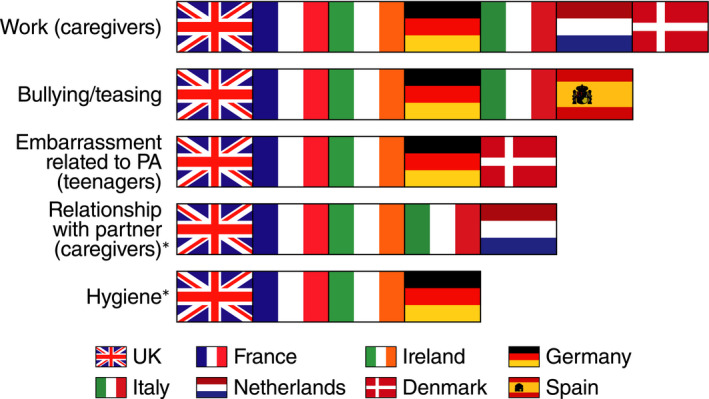
Country differences: concepts raised or endorsed by country. *Concepts were reported spontaneously, not probed for. PA, peanut allergy; UK, United Kingdom. The figure shows concepts that were endorsed or reported in some (not all) countries, for example participants in all countries were asked about peanut allergy–related bullying or teasing history, which was endorsed as an issue in six of eight countries. Asterisked concepts were not probed for in every country; the figure identifies countries where asterisked concepts were spontaneously raised by participants

The main themes in the conceptual models were reported by participants from all countries, with the exception of the impact on work, which was not reported by caregivers in Spain. Among other inter‐country differences, the aspect of PA avoidance involving hygiene (eg handwashing and asking others to wash their hands) was reported by participants only in Germany, France, the United Kingdom and Ireland, while the impact on the caregiver's relationship with their partner was not discussed by participants in Germany, Spain or Denmark. Embarrassment was not reported by teenagers in Italy, Spain or the Netherlands; bullying or teasing was not reported in the Netherlands or Denmark (Figure 5).

### 3.3 Conceptual models

The conceptual models illustrate the relationships between the main coping strategies and their impact in coping with PA (Figures 3 and 4). Having PA means that participants must avoid peanuts due to fear of a reaction and implement various strategies to cope with and control this risk. These coping behaviours can affect health‐related quality of life (social/work activities, relationships and emotions).

The colour spectrum between coping and control and the impacts demonstrates the spectrum of behaviours reported by participants, ranging from either a highly vigilant approach or a careless approach, with the middle section indicating a more positive or neutral impact on other concepts. The two‐way arrows between coping and control and the impacts show that their influences go both ways; for example, the level of anxiety experienced by an individual can mean that a person is more vigilant, compared to an individual with a low level of anxiety.

The extent to which participants are impacted by coping and control behaviours is influenced by several key themes, or moderators, which, for children and teenagers, included their levels of independence and self‐responsibility. Key moderators for caregivers were their levels of control over the child's food and environment. The attitudes and awareness levels of others (non‐family) towards PA were important moderators for both children/teenagers and caregivers. Other identified moderators (some shown in Figures 3 and 4) could have positive or negative impacts depending on other factors such as whether a school environment is peanut‐free or not; child age; perceived severity and sensitivity of PA; level of certainty regarding control of PA; number of past reactions; severity of past reactions (mild, moderate or severe); and how reactions were treated.

Coping and control strategies—and level of confidence in the effectiveness of these strategies—may influence whether a reaction occurs, which in turn may influence future coping and control behaviours. However, it was not clear whether the perception among participants of the severity of previous reactions to PA influenced their coping strategy.

Results from APPEAL‐2 can be viewed along with learnings by age group in the interactive and video content included in the Appendix S1: Video S1.

### 3.4 Subgroup analysis for participants with peanut allergy only (no other food allergies)

All the main themes in the conceptual model were all reported by participants with PA only, and no additional food allergies. All sub‐themes or concepts discussed in the results section were reported spontaneously by this group, with the exception of bullying or teasing which was reported only after prompting by the teenage participants with PA only (eight participants). This may be owing to the sensitivity of the topic. In addition, the concept of “responsibility” was not reported spontaneously by the children (≤12 years) in this subgroup (10 participants). However, teenagers in the sample generally discussed “responsibility” more readily as a whole, which may reflect the relevance of the concept to this age group. Caregivers of children with PA only and no additional food allergies spontaneously reported all of the themes and sub‐themes discussed in the results section. The subgroup of participants with PA only includes participants reporting a very minimal impact of PA on their lives as well as some in each age group reporting a substantial burden.

## 4 DISCUSSION

This large qualitative study is the first to highlight the specific burden of PA on individuals and their caregivers across eight European countries at one time, using conceptual models to illustrate the relationships between the main themes. Novel findings relate to the real‐world effects on patients and families and of the type of coping strategies used. Central themes described the fear of a reaction and the coping strategies needed to control that fear and maintain safety. The resulting stress may lead to anxiety and avoidance or frustration and risky behaviours, with direct and indirect effects on emotional adjustment, food choice, social interaction, development, confidence and overall quality of life. Moderating factors such as attitudes and awareness can increase or diminish this impact.

These results support previous findings from qualitative research in PA and provide unique insights. Some PA‐specific impacts reported here were noted in a smaller qualitative study of families of children with PA,^9^ including children feeling excluded or different and being teased, the social challenges of eating in restaurants, and the lack of awareness of PA on the part of restaurant staff and others. Moderating factors in the caregiver conceptual model identified in APPEAL‐2 are reflected in previously reported observations in qualitative studies of PA and/or food allergy. These include the burden of meal preparation, ensuring the home is nut‐free,^9^ and difficulties with teenagers’ transitions to independence and the diminishment of caregiver control.^10^, ^21^

Although many of the main concepts described here have been reported previously in food allergy studies, it is important that these are confirmed specifically for PA.^21^, ^22^ Previous qualitative research documented caregivers’ fear and hesitation related to AAI use,^21^ which was also reported by teenagers and caregivers in the current study. A large qualitative study of food allergy described how fear of a reaction drove particular coping strategies, conceptualized on a continuum from maximization (extreme avoidance) to minimization (risky behaviours).^7^ In APPEAL‐2, the colour spectrum in the conceptual models illustrates a comparable concept.

The conceptual models help illustrate the wide‐ranging impacts of coping strategies required when living with PA and provide nuanced and novel insights into how coping strategies may adversely impact mental well‐being and everyday life. For example, the use of “avoidance” has social and emotional consequences for children and teens, and may impact normal development. The use of “hygiene”—involving frequent handwashing and having to urge others to wash their hands before any potential contact—was discussed spontaneously by approximately a quarter of children and teenagers or their caregivers. This exemplifies how just one aspect of peanut vigilance/avoidance can impact social interactions, activities and relationships. In addition, over a third of caregivers reported that their child's PA had a negative impact on their work or career, including having to take time off and decreasing their working hours, demonstrating the potential socioeconomic impacts of PA.

Although the reported degree of impact of PA varied among participants, no clear link was identified between past history of PA (such as severity of reaction) and perceptions of the impact of PA. This illustrates the importance of psychological as well as clinical variables. The absence of such a link may be due to a reduction in uncertainty when a reaction is experienced and is successfully managed,^23^ or to other moderators, such as levels of social support and awareness. These findings will be further investigated using combined data from APPEAL‐1 and 2 and quantitative analysis. Additional longitudinal research is also needed to reveal how these variables are temporally related. The subgroup analysis also showed that participants with PA and those with multiple food allergies reported similar themes in the conceptual models. To the knowledge of the authors, this is a novel insight not previously identified in quantitiative studies. These results support and extend the results of a study in families with peanut‐allergic children, which found no differences between participants with PA only and those with multiple food allergies in allergy‐related impact on quality of life or anxiety for children or their parents/caregivers.^2^

Our results identify key opportunities to reduce the burden of living and coping with PA. The central moderating role of “other people” in the conceptual models demonstrates the importance of increasing awareness and understanding of PA in both the general public and healthcare professionals across Europe. More training and information on AAI use for adolescents and parents of children and adolescents could reduce the fear of—and increase confidence in—administering AAIs.^24^, ^25^ The uncertainty around precautionary allergen labelling highlights the need for regulations requiring clearer and more meaningful precautionary allergen labelling in Europe^26^, ^27^ and for development of more informative communication around food allergen risk and safety in general.

The APPEAL‐1 and APPEAL‐2 studies contribute to a clearer understanding of the relationship between coping strategies and the diverse psychosocial impacts of PA, which may facilitate the design of optimally effective interventions. These findings may also help identify subgroups of patients and caregivers who may benefit from emerging treatments or need additional support, as well as the factors that impact therapeutic outcomes.

Some limitations should be considered when interpreting the results of APPEAL‐2. The study required recruitment minimums of 50% of participants reporting severe PA and 25% who had used an AAI or experienced a life‐threatening event. Therefore, all participants had a self‐ or caregiver‐reported “moderate” or “severe” PA, thus omitting individuals who perceived a milder impact. Only children and teenagers with PA were included; the experience of adults with PA was not studied. The caregiver sample was almost all female, thus underrepresenting male caregiver experience, and small numbers of participants in some countries limited inter‐country comparison. Different interview methodologies (face‐to‐face and telephone) were used, which may have affected data quality; however, the length of interview was the same across methods, and the same concepts were revealed by participants in both face‐to‐face and telephone interviews.

In conclusion, this large European qualitative study highlights the varied and often substantial impact of PA on children, teenagers and caregivers. The two conceptual models illustrate how strategies related to coping and control are driven by the fear of PA reactions, and the emotional, social, relationship and work impacts that stem from this fear.

These results suggest that the current standard of PA care and support is insufficient and in need of several improvements. Attention to psychological and developmental factors, as well as the moderating role of environmental variables such as community and socioeconomic factors, is vital to improve the PA care model. Specific needs include improved regulation of food labelling; better education about PA for the general public; more information for patients and caregivers (including the causes and mechanisms of allergic reactions and the use of AAIs for emergencies); integration of psychological services in food allergy clinics; and, potentially, treatments that can reduce the risks of PA and thus alleviate its impacts. Creative qualitative and quantitative research approaches will enable improved modelling of the costs, risks and benefits of any treatment. The qualitative findings in the APPEAL‐2 study can help to broaden and enrich the knowledge base for future PA studies.

## CONFLICTS OF INTEREST

ADG reports lecture honoraria/consultation fees from Aimmune Therapeutics and research support from National Children's Research Centre, Our Lady's Children's Hospital, Crumlin, Dublin 12. KG and SA are consultants to Aimmune Therapeutics. FT is chair of the EAACI Patient Organizations Committee and member of Team APPEAL; the national patient advocacy organization has received honoraria from Aimmune Therapeutics. LR, SS, MP, AS, PC and BH are members of Team APPEAL whose patient advocacy organizations have received honoraria from Aimmune Therapeutics. RR and AV are employees of Aimmune Therapeutics. MF is a member of Team APPEAL and has received honoraria from Aimmune Therapeutics for advice; honoraria from Nutricia; research funding from NIAID, NIH, UK FSA, FARE, MRC and Asthma UK Centre, UK Department of Health through NIHR, National Peanut Board, Osem. HRF is a member of Team APPEAL and reports honoraria from Aimmune Therapeutics. KB reports consulting for Aimmune Therapeutics, DBV Technologies, Bencard Allergie and HAL Allergy; speakers bureau for Aimmune Therapeutics, DBV Technologies, HAL Allergy, Nutricia, Thermo Fisher Scientific, ALK, Allergopharma and Nestlé, and conducting clinical trials for Aimmune Therapeutics, DBV Technologies and Hipp. MFR reports consultancies for Aimmune Therapeutics, DBV, Novartis and Schreiber Foods; research funding from European Commission, MINECO and ISCIII (Spanish government); speakers bureau for ALK, Allergy Therapeutics, Diater, Fundacion SEAIC, HAL Allergy and Thermo Fisher Scientific.

## Supporting information

Appendix S1

## Data Availability

The data that support the findings of this study captured in the current article are available from the corresponding author upon reasonable request.
