# Supplementary material for: APPEAL‐2: A pan‐European qualitative study to explore the burden of peanut‐allergic children, teenagers and their caregivers
Source: Clin Exp Allergy. 2020 Sep 15;50(11):1238–48. doi: 10.1111/cea.13719 (PMC7780296; doi:10.1111/cea.13719)
Supplement: Supplementary file 1 — Appendix S1 [file CEA-50-1238-s001.docx]

**Supplementary Materials**

**Background form: children (parent-completed)**

1. How old is your child? _____
2. Is your child…
   - Male
   - Female
   - Other
   - Prefer not to answer
3. Is your child…
   - White British/ other
   - Black British/ Caribbean/ African/ other
   - Asian Indian/ Pakistani/ Bangladeshi/ Other
   - Chinese
   - Mixed race
   - Other:
   - Prefer not to answer
4. How old was your child when they had their first allergic reaction to peanut? ______
   - Don’t know
5. How old was your child when their peanut allergy was diagnosed by a medical doctor/nurse? _______
   - Don’t know
6. Is your child allergic to any food other than peanut?
   - If yes, which foods: ______________________
7. Not including allergies, does your child have any other illnesses or conditions which limit their daily activities?
   - If yes, what condition/s: ___________________
8. Do any other members of your family have peanut allergy?

If yes:

- - The child’s mother
  - The child’s father
  - The child’s brother or sister
  - Other __________

1. Has your child been prescribed an adrenaline-autoinjector (e.g. Epi-pen) for their peanut allergy?
   - Yes
   - No
   - Don’t know
2. How confident does your child feel in managing their reactions to peanut? *Please give your impression of* ***how confident your child feels****.*
   - Not at all confident
   - Somewhat confident
   - Confident
   - Very confident
   - Don’t know
3. How much control does your child feel they have over their peanut allergy? *Please give your impression of* ***how much control your child feels****.*
   - No or very little control
   - Some control
   - A good amount of control
   - Full control
   - Don’t know

**Background form: adolescents**

1. What is your age? _____
2. Are you…
   - Male
   - Female
   - Other
   - Prefer not to answer
3. Are you…
   - White British/ other
   - Black British/ Caribbean/ African/ other
   - Asian Indian/ Pakistani/ Bangladeshi/ Other
   - Chinese
   - Mixed race
   - Other:
   - Prefer not to answer
4. How old were you when you had your first allergic reaction to peanut? _______
   - Don’t know
5. How old were you when your peanut allergy was diagnosed by a medical doctor/nurse? ____
   - Don’t know
6. Are you allergic to any food other than peanut?
   - If yes, which foods: ______________________
7. Not including allergies, do you have any other illnesses or conditions which limit your daily activities?
   - If yes, what condition/s: ___________________
8. Do any other members of your family have peanut allergy?

If yes:

- - Mother
  - Father
  - Brother or Sister
  - Other __________

1. Have you been prescribed an adrenaline-autoinjector (e.g. Epi-pen, Jext) for your peanut allergy?
   - Yes
   - No
   - Don’t know
2. How confident do you feel in managing your reactions to peanut?
   - Not at all confident
   - Somewhat confident
   - Confident
   - Very confident
3. How much control do you feel you have over your peanut allergy?
   - No or very little control
   - Some control
   - A good amount of control
   - Full control

**Background form: parents as caregiver participants**

1. What is your age? _____
2. Are you…
   - Male
   - Female
   - Other
   - Prefer not to answer
3. What is your child’s age? _____ ***(if more than 1 child with Peanut Allergy please think about the child with the most severe allergy / most impacted by their allergy for the purpose of this study)***
4. Is your child…
   - Male
   - Female
   - Other
   - Prefer not to answer
5. Is your child…
   - White British/ other
   - Black British/ Caribbean/ African/ other
   - Asian Indian/ Pakistani/ Bangladeshi/ Other
   - Chinese
   - Mixed race
   - Other:
   - Prefer not to answer
6. How old was your child when they had their first allergic reaction to peanut? ______
   - Don’t know
7. How old was your child when their peanut allergy was diagnosed by a medical doctor/nurse? ______
   - Don’t know
8. Is your child allergic to any food other than peanut?
   - If yes, which foods: ______________________
9. Not including allergies, does your child have any other illnesses or conditions which limit their daily activities?
   - If yes, what condition/s: ___________________
10. Do any other members of the family have peanut allergy?

If yes:

- - Participant
  - Partner / Spouse
  - Other child/ children
  - Other ________

1. Has your child been prescribed an adrenaline-autoinjector (e.g. Epi-pen) for their peanut allergy?
   - Yes
   - No
   - Don’t know
2. How confident do you feel in managing your child’s reactions to peanut?
   - Not at all confident
   - Somewhat confident
   - Confident
   - Very confident
3. How much control do you feel you have over your child’s peanut allergy?
   - No or very little control
   - Some control
   - A good amount of control
   - Full control

**Interview guide: children**

- **INSTRUCTIONS TO INTERVIEWER (NOT TO BE READ TO THE PARTICIPANT)**
- The guide is intended to be used as the basis of discussion, but where possible the interview should feel like a conversation.
- Not all questions and probes need to be asked – throughout interview, only ask questions/use probes where appropriate, taking into account what has already been discussed, as well as the clinical profile of the participant.
- Additional unscripted probes to be used to gain further information or clarification may include: Clarification (That sounds interesting – can you explain further? I don’t quite understand that); Expressing understanding (How did you cope with that?); Justification (Can you tell me a bit more about that? Why do you think X is… [e.g. important/unimportant]?); Relationships (Could you explain how you feel/think these two things are linked?); Extending narrative (Tell me a bit more about that); Accuracy (Let’s see if I’ve got that right).
- Make notes so that you can return to topics later in the interview and avoid repetition.
- Instructions to the interviewer are in bold and brackets **[like this]** – do not read this text to the participant.

Good (morning/afternoon/evening), [introduce self].

Thank you for taking part in this interview. I will be talking with you about your peanut allergy today to help us understand how your peanut allergy affects your life.

Our conversation will be recorded so that we can accurately represent what you are saying during the discussion in our research report. We will now begin recording. OK?

*(If participant does not wish to be recorded, interviewer should not proceed with the interview)*

**START RECORDING**

***Begin recorder:*** Today is [date]. This is participant ID [insert number here].

- Do I have your permission to record this interview? [YES/NO]

Everything you say is confidential. You do not have to answer any questions you do not want to answer, you can take a break or stop the interview, and you may ask me questions at any time.

Do you have any questions before we begin? [YES/NO] If yes, please ask the participant to elaborate.

[IF PARENT IS STILL PRESENT, PARENT TO LEAVE THE ROOM NOW]

**Background**

First, I would like to ask you some questions to give us a general picture of your experience with peanut allergy.

1. How old were you when you first knew about your peanut allergy?
2. How did you find out you had a peanut allergy?
3. How many times have you had an allergic reaction to peanut?
   1. Can you tell me what happened last time?
      1. Where were you? Who were you with?
      2. How did you feel at the time? What happened afterwards? Did you need to take pills/get an injection/jab/go to hospital?
      3. How did you feel afterwards?
      4. Do you remember what you ate that caused the reaction?
4. What do you find most difficult about having a peanut allergy?
   1. What do you dislike about it?

**[NOTE: if ‘uncertainty’ is reported, follow up: ‘What do you mean by ‘uncertainty’? ‘In what way does uncertainty affect you?’]**

**Daily activities**

I would like to understand how living with peanut allergy impacts your day-to-day life.

1. Can you talk me through a typical day for you, thinking about what you normally do whether on a school day or a weekend/holiday, and how your peanut allergy affects the things you do?

**[PROBE: for example, breakfast, lunch at school, dinner, where you go, what you do, how you behave, school, activities/sports, friendships, how well you sleep]**

1. What are the main ways your peanut allergy affects your day-to-day life?

**Coping and support**

1. How confident are you that you would know when you have an allergic reaction to peanut?
2. If prescribed AAI: Do you know when to use your AAI (i.e. an adrenaline pen)?
3. Have you been shown how to use it? Did you find this helpful? why?
   - 1. If yes: who by?
4. How often do you carry your AAI with you?
5. Do you forget your AAI?
   - 1. If yes: how do you feel about that?
6. How confident are you talking to people about your peanut allergy? **[PROBE: people you know vs. people you do not know]**
   1. If confident: how do you talk/what do you say?
   2. If not very confident: why not?
7. Do you feel in control of your peanut allergy?
8. Do you get support a) at home; b) at school; c) from friends?

**Emotional impact**

1. How does having peanut allergy make you feel?

**[INTERVIEWER: note all emotions experienced]**

For each emotion:

- 1. What makes you feel [emotion]?

**[PROBE: is it due to reactions, needing to avoid peanuts, feeling scared of having a reaction, feeling that you are different?]**

- 1. How does feeling [emotion] affect your life? (e.g. how does it affect what you do, or what does it stop you doing)

**if not spontaneously reported, ask does your peanut allergy ever make you feel: worried, scared, lonely/isolated, frustrated, sad, guilty, stressed, uncertain/unsure?**

**[NOTE: if ‘uncertainty’ is reported, follow up: ‘What do you mean by ‘uncertainty’? ‘In what way does uncertainty affect you?’]**

- 1. If yes for any of the above: Why do you feel that way?

1. Do you feel that you are different from other children your age because of your peanut allergy?
   1. If yes: in what way?
   2. How does this affect you?
   3. How does this make you feel?
2. Do you feel that you are treated differently because of your peanut allergy?
3. By who? In what way?
4. How does this make you feel?
5. Have you ever been teased or picked on because of your peanut allergy?
   1. If yes: can you tell me what happened?
   2. How often does this happen?
   3. How does this make you feel?
   4. Who do you talk to or tell about this?

**Social and educational impact**

1. Can you tell me about any ways that peanut allergy affects a) your time at school and your school work; b) things you do with friends; c) things you like to do or hobbies; d) you and your family’s holidays and how you travel?

**[PROBES:]**

- **lunch times at school, sitting with friends/classmates**
- **missing school/activities, feeling left out of activities, not being allowed to take part in activities**
- **impact on school work or performance**
- **being treated differently and by whom**

1. Why? How often? What type of activities/events?
2. How does this impact you?
3. How does it make you feel?
4. Do you think your parents supervise you more than your friends’ parents?
5. If yes: why? In what way?
6. If no: did they when you were younger?
   - 1. If yes: why? In what way?
7. Do they come with you to parties or to your friends’ houses because of your peanut allergy?
8. Can you tell me about any ways your peanut allergy affects the things you do with your family? For example, going for days out or going on holiday
9. In what way?
10. Who – brothers/sisters, mum, dad?
11. How does this make you feel?
12. Does your peanut allergy affect how you get on with your family?
13. In what way?
14. Who – brothers/sisters, mum, dad?
15. How does this make you feel?

**Wrap up**

1. Of all the ways we have discussed peanut allergy has impacted your life, what do you feel impacts you the most?
2. Why?
3. How would you like this to change?
4. Are there any other ways in which your life is, or has been, impacted by peanut allergy that we have not talked about?

**Thank you for your helpful feedback.**

**We really appreciate the time you have taken to participate in this study.**

**Interview guide: adolescents**

**INSTRUCTIONS TO INTERVIEWER (NOT TO BE READ TO THE PARTICIPANT)**

- The guide is intended to be used as the basis of discussion, but where possible the interview should feel like a conversation.
- Not all questions and probes need to be asked – throughout interview, only ask questions/use probes where appropriate, taking into account what has already been discussed, as well as the clinical profile of the participant.
- Additional unscripted probes to be used to gain further information or clarification may include: Clarification (That sounds interesting – can you explain further? I don’t quite understand that); Expressing understanding (How did you cope with that?); Justification (Can you tell me a bit more about that? Why do you think X is… (e.g. important/unimportant)?); Relationships (Could you explain how you feel/think these two things are linked?); Extending narrative (Tell me a bit more about that); Accuracy (Let’s see if I’ve got that right).
- Make notes so that you can return to topics later in the interview and avoid repetition
- Instructions to the interviewer are in bold and brackets **[like this]** – do not read this text to the participant

Good (morning/afternoon/evening), introduce self.

Thank you for taking the time to participate in this interview. I will be talking with you about your peanut allergy to help us and the sponsor of this study, Aimmune Therapeutics, to understand the different ways in which peanut allergy impacts people’s lives.

Our conversation will be recorded so that we can accurately represent what you are saying during the discussion in our research report. We will now begin recording. OK?

*(If participant does not wish to be recorded, interviewer should not proceed with the interview)*

**START RECORDING**

***Begin Recorder:*** Today is [date]. This is participant ID [Insert number here]

- Do I have your permission to record this interview? [YES/NO]

I’m first going to cover a few points to make sure you understand what we will discuss and your rights as a research participant:

- Please could you confirm you understand this interview is about your experiences with peanut allergy to help the researchers and the sponsor of this study, Aimmune Therapeutics, to understand the impact of peanut allergy on people’s lives? [YES/NO]
- I may use your first name in the interview, but in reports no names will be attached to any comments. Your name and any other personally identifiable information will be removed and stored separately. Please could you confirm you understand that your data will remain confidential? [YES/NO]
- You may also request for your data to be removed at any time. We will remove the data unless we have already destroyed your personal identifiable information at the time of request. Please could you confirm you understand you can request to remove your data? [YES/NO]
- Participation in this research is completely voluntary. You do not have to answer any questions you do not wish to answer, and you can stop the interview. Please could you confirm you understand that taking part in this interview is voluntary? [YES/NO]

Finally, before we start the interview, a few things to make sure our discussion is as productive as possible:

- My role here is to ask questions and to listen. I will be taking notes and will also verbally summarize what you tell me at times to ensure I understand what you mean.
- I will move the discussion from one question to the next to try to keep us on track so that we can finish on time. Some of my questions may seem repetitive but I will try to keep that to a minimum.
- I am not a medical doctor, so I am not qualified to give medical advice. If you have any questions about your peanut allergy as a result of our conversation today, I advise you to follow up with your regular doctor.
- During the interview we will ask you about how you feel or think about your experiences, there are no right or wrong answers, we understand that everyone has different experiences and we are interested to hear what you have to say.
- Please speak loudly enough to be heard for the recording.
- Do you have any questions before we begin? [YES/NO] [If yes, please ask the participant to elaborate.]
- [IF PARENT IS STILL PRESENT, PARENT TO LEAVE THE ROOM NOW]

**Background**

First, I would like to ask you some questions to give us a general picture of your experience with peanut allergy.

1. How old were you when you were first aware of your peanut allergy?
2. How severe do you think your peanut allergy is? **[PROBE: mild/moderate/severe]**
   1. Why do you rate it as [mild/moderate/severe]?
3. How many times have you had a reaction to peanut in your daily life? (not a food challenge in a clinical setting)
   1. How frequently do you have reactions to peanut?
   2. How severe are your typical reactions? **[PROBE: mild/moderate/severe]**
      1. What symptoms do you experience?
      2. Do you manage them yourself?
      3. Do you usually require treatment? If yes, what type? Adrenaline injection, antihistamine?
   3. Have you ever had to go to hospital or stay in hospital due to a reaction to peanut?
   4. How severe was your WORST reaction to peanut? **[PROBE: mild/moderate/ severe]** Can you describe what happened? **(PROBE below items if not addressed)**
      1. What symptoms did you experience?
      2. Did you manage it yourself?
      3. Did you require any treatment? If yes, what type?
      4. How did the experience make you feel?
      5. Did the experience have any impact on you? **[PROBE: e.g. emotional, practical (educational, coping, communication), daily life]**
      6. Do you remember the specific food that caused the reaction?
   5. When was your most RECENT reaction to peanut? **[if not their worst experience]** Can you describe what happened? **(PROBE below items if not addressed)**
      1. What symptoms did you experience?
      2. Did you manage it yourself?
      3. Did you require any treatment? If yes, what type?
      4. Do you remember the specific food that caused the reaction?
4. What is the most difficult part of having a peanut allergy?
   1. What do you dislike about it?

**[NOTE: if ‘uncertainty’ is reported, follow up: ‘What do you mean by ‘uncertainty’? ‘In what way does uncertainty affect you?’]**

**Daily activities**

I would like to understand how living with peanut allergy impacts your day-to-day life.

1. Can you talk me through a typical day for you, thinking about your daily routines and activities whether on a school day or a weekend/holiday and how your peanut allergy affects the things you do?

**[PROBE: for example, breakfast or planning lunch, packing your school bag, where you go, what you do, how you behave, school, (after-school) activities/sports, friendships/romantic relationships, evening routine, how well you sleep]**

1. What are the main ways your peanut allergy impacts your day-to-day life?
2. Can you tell me about things you have to do in your day-to-day life to ensure you are not exposed to peanuts?

**[PROBE: planning, avoiding high-risk environments, communicating PA to other people]**

- 1. What type of things do you need to do? For what events/activities?
  2. How much time/effort does this take?
  3. How does this make you feel?

1. Do you find that your peanut allergy affects your choices or holds you back when…?
   1. Eating out at restaurants/cafes
   2. Buying food
   3. School/job options
   4. Socialising/special occasions
   5. Dating
   6. Travelling on public transport

If restricted choices are discussed:

How do you think this compares to what other people your age can do?

**Coping and support**

1. How would you say you cope with your peanut allergy at present?

**[PROBE: What do you do, or what have you done, to make living with PA easier or more manageable? E.g. carry AAI, tell friends/teachers/classmates, avoid high-risk situations]**

Is coping easier in certain situations? If so, which are easier, and which are hardest?

1. How would you say you coped with your peanut allergy when it was first diagnosed?
   1. What have you learned about coping or living with peanut allergy?
2. How confident are you in recognising the symptoms of an allergic reaction to peanut?
   1. Which symptoms would prompt you to call home/ 999, take antihistamine, use an AAI or wait and see)?
   2. If prescribed AAI: how confident are you that you know when to use your AAI? When would you know to use? e.g. certain symptoms would prompt.
   3. Did you receive training in how to use it?
      1. If yes: who from? Did you find it useful for you? why?
   4. How often do you carry your AAI with you?
   5. How do you feel if you forget your AAI?
      1. How does it impact you and your day?
3. How confident are you talking to people about your peanut allergy?

**[PROBE: familiar vs. unfamiliar people]**

1. How much in control do you feel you are over your peanut allergy?
2. How much support do you feel your doctor or allergy specialist provides to help you with your peanut allergy?
3. How much support do you feel your school provides to help you with your peanut allergy?
4. How satisfied are you with advice or information you have received from healthcare professionals about your peanut allergy?
5. Have you ever sought information or advice from a patient group website or helpline?
   1. Have you ever looked for information about peanut allergy online?
      1. If yes: did you find the information you were looking for?
      2. Was this helpful?
      3. IF YES: In what way?

**Other people’s understanding of PA**

1. How do people who do not have peanut allergy view it?
   1. Do you think other people understand what it’s like to have a peanut allergy?
2. Do you feel that your peanut allergy and how it impacts your life is taken seriously?
   1. If yes: Who takes it seriously? **[PROBE: general public, family, friends, teachers, doctors]** In what way(s)? what effect does this have on you?

If no: Who doesn’t take it seriously? **[PROBE: general public, family, friends, teachers, doctors]** In what way(s)? what effect does this have on you?

**Independence**

1. Do you feel you have as much, more or less independence compared with other people your age?
   1. If less: in what way? Why?
2. Do you feel your parents supervise you as much as, more than, or less than parents of other people your age?
3. If more: why? In what way?
4. If as much/less: did they supervise you more when you were younger?
   - 1. If yes: why? In what way?

**Emotional impact**

1. How does having peanut allergy make you feel?

**[INTERVIEWER: note all emotions experienced]**

For each emotion:

- - 1. What makes you feel [emotion]?

**[PROBE: is it due to reactions, needing to avoid peanuts, feeling scared of having a reaction, feeling that you are different from others?]**

- - 1. How does feeling [emotion] impact your life?

**[PROBE: Does it impact your social activities, daily activities or leisure activities?]**

- - 1. How often do you feel [emotion]?

**if not spontaneously reported, ask does your peanut allergy ever make you feel: worried/anxious, scared, self-conscious, isolated/lonely, frustrated, sad, guilty, stressed, uncertain/unsure?**

**[NOTE: if ‘uncertainty’ is reported, follow up: ‘What do you mean by ‘uncertainty’? ‘In what way does uncertainty affect you?’]**

1. If yes for any of the above: What makes you feel [emotion]?

**[PROBE: Is it due to reactions, needing to avoid peanuts, feeling scared of having a reaction, how other people treat you because of your PA?]**

1. How does feeling [emotion] impact your life?

**[PROBE: Does it impact your daily activities, social activities or leisure activities?]**

1. Do you feel that your life is different from others your age because of your peanut allergy?
2. If yes: in what way?
3. How does this impact your life?
4. How does this make you feel?
5. Do you feel that you are treated differently because of your peanut allergy?
6. By who/where [PROBE: home, school, with friends]? In what way?
7. How does this impact you?

**[PROBE if relevant: How does this impact your relationships with family (parents, brothers/sisters) or friends?]**

1. Have you ever been treated unkindly because of your peanut allergy?
2. If yes: can you tell me what happened?
3. How often does this happen?
4. How does this impact you?
5. What support do you have?
   - 1. If none; who do you talk to or tell about these experiences?
     2. If no one: why?

**Social and educational impact**

1. Does having peanut allergy impact a) school activities and performance at school; b) social activities; c) leisure activities (incl. holidays and travel, e.g. flying) or hobbies? **[REPEAT FOR EACH TYPE OF ACTIVITY]**
2. Why? How often? What type of activities/events?
3. How does it impact you? Do you avoid it? Or did you have to miss out on it?
4. How does it make you feel?
5. Does having peanut allergy impact your relationships with other people?

**[PROBE: teachers, classmates, friends, boyfriends/girlfriends, parents, siblings, new people]**

1. In what way(s)?
2. Who?
3. How does this make you feel?

**[FURTHER PROBES:**

1. **Impact on activities they do with other people**
2. **Feeling left out of activities other people are doing**
3. **Feeling uncomfortable going out with friends or going to their houses**
4. **Feeling isolated**
5. **Experiences with other people being insensitive**
6. **Embarrassing to have to tell people about PA (including staff in restaurants)]**
7. Do your parents ever **not** allow you to go somewhere because of your peanut allergy?
8. If yes: Why? How often? What type of events/activities?
9. If no: Did they when you were younger? (probe questions above)
10. How does/did this make you feel?

**[PROBE: impact on friendships; impact on relationship with parents/family]**

**Treatment**

1. What would a successful treatment for peanut allergy mean for you?
2. What would you want it to do?
3. What change would this make to your life?
4. Of all the ways we have discussed peanut allergy has impacted your life, what do you feel impacts you the most?
5. Why?
6. How would you like this to change?
7. Do you have allergies other than peanut allergy?
8. If yes: what would a successful treatment for peanut allergy mean for you if you still had your other allergies?

**Wrap up**

1. Are there any other ways in which your quality of life is, or has been, impacted by peanut allergy that we have not discussed?

**Thank you for your helpful feedback.**

**We really appreciate the time you have taken to participate in this study.**

**Interview guide: parents as caregiver participants**

- **INSTRUCTIONS TO INTERVIEWER (NOT TO BE READ TO THE PARTICIPANT)**
- The guide is intended to be used as the basis of discussion, but where possible the interview should feel like a conversation
- Not all questions and probes need to be asked - throughout interview, only ask questions/use probes where appropriate, taking into account what has already been discussed, as well as the clinical profile of the participant.
- Additional unscripted probes to be used to gain further information or clarification may include: Clarification (That sounds interesting – can you explain further? I don’t quite understand that); Expressing understanding (How did you cope with that?); Justification (Can you tell me a bit more about that? Why do you think X is… (e.g. important/unimportant)?); Relationships (Could you explain how you feel/think these two things are linked?); Extending narrative (Tell me a bit more about that); Accuracy (Let’s see if I’ve got that right).
- Make notes so that you can return to topics later in the interview and avoid repetition
- Instructions to the interviewer are in bold and brackets **[like this]** – do not read this text to the participant

Good (morning/afternoon/evening), introduce self.

Thank you for taking the time to participate in this interview. I will be talking with you about your child’s peanut allergy to help us and the sponsor of this study, Aimmune Therapeutics, to understand the different ways in which peanut allergy impacts people’s lives.

Our conversation will be recorded so that we can accurately represent what you are saying during the discussion in our research report. We will now begin recording. OK?

*(If participant does not wish to be recorded, interviewer should not proceed with the interview)*

**START RECORDING**

***Begin Recorder:*** Today is [date]. This is participant ID [Insert number here]

- Do I have your permission to record this interview? [YES/NO]

I’m first going to cover a few points to make sure you understand what we will discuss and your rights as a research participant:

- Please could you confirm you understand this interview is about your experiences with your child’s peanut allergy to help the researchers and the sponsor of this study, Aimmune Therapeutics, to understand the impact of peanut allergy on people’s lives? [YES/NO]
- I may use your or your child’s first name in the interview, but in reports no names will be attached to any comments. Your name and any other personally identifiable information will be removed and stored separately. Please could you confirm you understand that your data will remain confidential? [YES/NO]
- You may also request for your data to be removed at any time. We will remove the data unless we have already destroyed your personal identifiable information at the time of request. Please could you confirm you understand you can request to remove your data? [YES/NO]
- There are no anticipated physical risks associated with your participation. Any information you provide will help us in understanding the research topic but may be of no direct benefit to you. Please could you confirm you understand the risks and benefits of participating in this research? [YES/NO]
- Participation in this research is completely voluntary. You do not have to answer any questions you do not wish to answer, and you can stop the interview. Please could you confirm you understand that taking part in this interview is voluntary? [YES/NO]

Finally, before we start the interview, a few things to make sure our discussion is as productive as possible:

- My role here is to ask questions and to listen. I will be taking notes and will also verbally summarize what you tell me at times to ensure I understand what you mean.
- I will move the discussion from one question to the next to try to keep us on track so that we can finish on time. Some of my questions may seem repetitive but I will try to keep that to a minimum.
- I am not a medical doctor, so I am not qualified to give medical advice. If you have any questions about your peanut allergy as a result of our conversation today I advise you to follow up with your regular doctor.
- During the interview we will ask you about how you feel or think about your experiences, there are no right or wrong answers, we understand that everyone has different experiences and we are interested to hear what you have to say.
- Please speak loudly enough to be heard for the recording.
- Do you have any questions before we begin? [YES/NO] If yes, please ask the participant to elaborate.

**Background**

First, I would like to ask you some questions to give us a general picture of your experience with peanut allergy.

1. How many children with peanut allergy do you have?

**If more than 1 (and/or if the participant has peanut allergy): Please think about the child with the most severe allergy, or most impacted by their allergy for the purpose of this interview. If you feel any issues discussed are impacted or experienced differently in your household because you have / because you have more than one child with an allergy, please point that out and tell us your thoughts.**

1. How old was your child when you first became aware of their peanut allergy?
2. How severe do you think your child’s peanut allergy is? **[PROBE: mild/moderate/severe]**
   1. Why do you rate it as [mild/moderate/severe]?
3. How many times has your child had a reaction to peanut in your daily life? (not a food challenge in a clinical setting)
   1. How frequently does your child have reactions to peanut?
   2. How severe are their typical reactions? **[PROBE: mild/moderate/severe]**
      1. What symptoms does your child experience?
      2. Does your child/do you manage them yourself?
      3. Does your child usually require treatment? If yes, what type? Adrenaline injection, antihistamine?
   3. Has your child ever had to go to hospital or stay in hospital due to a reaction to peanut?
   4. How severe was your child’s WORST reaction to peanut? **[PROBE: mild/moderate/ severe].** Can you describe what happened? **(PROBE below items if not addressed)**
      1. What symptoms did your child experience?
      2. Did your child/you manage it yourself?
      3. Did your child require any treatment? If yes, what type?
      4. How did the experience make you feel?
      5. Did the experience have any impact on you? **[PROBE: e.g. emotional, practical (work, coping, communication), daily life]**
      6. Do you remember the specific food that caused the reaction?
   5. When was your child’s most RECENT reaction to peanut?

**[if not their worst experience]** Can you describe what happened? **(PROBE below items if not addressed)**

- - 1. What symptoms did your child experience?
    2. Did your child/you manage it yourself?
    3. Did your child require any treatment? If yes, what type?
    4. Do you remember the specific food that caused the reaction?

1. What is the most difficult part of having child with a peanut allergy?

**[NOTE: if ‘uncertainty’ is reported, follow up: ‘What do you mean by ‘uncertainty’? ‘In what way does uncertainty affect you?’]**

**Daily activities**

I would like to understand how living with your child’s peanut allergy impacts your and their day-to-day life.

1. Can you talk me through a typical day for you, thinking about your and your family’s daily routines and activities whether on a weekday or a weekend/holiday and how your child’s peanut allergy affects the things you do?

**[PROBE: for example, breakfast or planning lunch, packing school bag, where you go, what you do, how you behave, work, school, (after-school) activities/sports, evening routine, how well you sleep]**

1. What are the main ways your child’s peanut allergy impacts a) your day-to-day life; b) their day-to-day life?
2. Can you tell me about things you or your child have to do in your day-to-day life to ensure your child is not exposed to peanuts?

**[PROBE: planning, avoiding high-risk environments, communicating PA to other people]**

- 1. What type of things do you need to do? For what events/activities?
  2. How much time/effort does this take?
  3. How does this make you feel?

1. Do you find that your child’s peanut allergy affects your or your child’s choices when…?
   1. Eating out at restaurants/cafes
   2. Buying food
   3. School/job options
   4. Socialising/special occasions
   5. Traveling on public transport

If restricted choices are discussed:

How do you think this compares to what other families/children your child’s age can do?

**Coping and support**

1. How would you say you and your child cope with their peanut allergy at present?

**[PROBE: What do you do, or what have you done, to make living with PA easier or more manageable? E.g. carry AAI, tell friends/teachers/classmates, avoid high-risk situations]**

Is coping easier in certain situations? If so, which are easier, and which are hardest?

1. How would you say you and your child coped with their peanut allergy when first diagnosed?
   1. What have you both learned about coping or living with their peanut allergy?
2. How confident are you and your child in recognising the symptoms of an allergic reaction to peanut?
   1. Which symptoms would lead you or your child to call home/999, provide/take antihistamine, use AAI or wait and see?
   2. If prescribed AAI: how confident are you that you know when to use an AAI?
   3. Did you both receive training in how to use it? Did you find it useful? why?
      1. If yes: who from?
   4. How often do you and your child carry an AAI with you?
   5. How do you both feel if you forget the AAI?
      1. How does it impact you and your day?
3. How much in control do you feel you are over your child’s peanut allergy?
4. How much support do you feel the doctor or allergy specialist provide to help you with their peanut allergy?
5. How much support do you feel your child’s school provides to help with their peanut allergy?
6. How satisfied are you with advice or information you have received from healthcare professionals about their peanut allergy?
7. Have you ever sought information or advice from a patient group website or helpline?
   1. Have you ever looked for information about peanut allergy online?
      1. If yes: did you find the information you were looking for?
      2. Was this helpful?
      3. IF YES: In what way?

**Emotional impact**

1. How does your child’s peanut allergy make a) you feel; b) them feel?

**[INTERVIEWER: note all emotions experienced]**

For each emotion:

1. What makes you/them feel [emotion]?

**[PROBE: is it due to reactions, needing to avoid peanuts, feeling scared of having a reaction?]**

1. How does feeling [emotion] impact your/their life?

**[PROBE: Does it impact your social activities, daily activities or leisure activities?]**

1. How often do you/they feel [emotion]?

**if not spontaneously reported, ask does your child’s peanut allergy ever make you/them feel: worried/anxious, scared, self-conscious, isolated/lonely, frustrated, sad, guilty, stressed, uncertain/unsure?**

**[NOTE: if ‘uncertainty’ is reported, follow up: ‘What do you mean by ‘uncertainty’? ‘In what way does uncertainty affect you?’]**

1. If yes for any of the above: What makes you/them feel [emotion]?

**[PROBE: Is it due to reactions, needing to avoid peanuts, feeling scared of having a reaction?]**

1. How does feeling [emotion] impact your/their life?

**[PROBE: Does it impact on daily activities, social activities or leisure activities?]**

1. Do you feel that a) your life; b) your child’s life is different from others/other children your child’s age because of their peanut allergy?
2. If yes: in what way?
3. How does this impact your/their life?
4. How does this make you/them feel?
5. Do you feel that your child is treated differently because of their peanut allergy?
6. By who/where [PROBE: home, school, friends]? In what way?
7. How does this impact them?

**[PROBE if relevant: How does this impact their relationships with family (parents, brothers/sisters) or friends?]**

1. Has your child ever been treated unkindly because of their peanut allergy?
2. If yes: can you tell me what happened?
3. How often does this happen?
4. How does this impact them?
5. What support do you/they have?

**Social, educational and family impact**

1. Does having your child’s peanut allergy impact a) your work activities, performance and career / their school activities and performance; b) your/their social activities; c) your/their leisure activities (incl. holidays and travel, e.g. flying) or hobbies? **[REPEAT EACH TYPE OF ACTIVITY FOR BOTH PARENT AND CHILD]**
2. Why? How often? What type of activities/events?
3. How does it impact you/them? Do you/they avoid it? Or did you/they have to miss out on it?
4. How does it make you/them feel?
5. Does having peanut allergy impact your child’s relationships with other people?

**[PROBE: teachers, classmates, friends, parents, brothers/sisters, other family members, new people]**

1. In what way(s)?
2. Who?
3. How does this make them feel?

**[FURTHER PROBES:**

1. **Impact on activities they do with other people**
2. **Feeling left out of, or actively excluded from, activities with other people**
3. **Feeling uncomfortable going out with friends or going to their houses**
4. **Feeling isolated**
5. **Experiences with other people being insensitive**
6. **Embarrassing to have to tell people about PA (including staff in restaurants)]**
7. Do you ever not let your child attend/participate in an event/activity because of their peanut allergy?
8. If yes: Why? How often? What type of events/activities?
9. If no: Did they when you were younger? (probe questions above)
10. How does/did this make you and your child feel?

**[PROBE: impact on friendships; impact on relationship with parents/family]**

1. Do you feel that you need to supervise your child more than other parents of children the same age?
2. Why?
3. How does this impact your child?
4. How does this make you and your child feel?

**[PROBE: impact on friendships; impact on relationship with parents/family]**

1. Does your child’s peanut allergy have an impact on other members of your family?
   1. In what way? Who – brothers/sisters?

**Treatment**

1. What would a successful treatment for peanut allergy mean for you and your child?
2. What would you want it to do?
3. What change would this make to your and their life?
4. Of all the ways we have discussed peanut allergy has impacted your and your child’s life, what do you feel impacts you and your child the most?
5. Why?
6. How would you like this to change?
7. Does your child have allergies other than peanut allergy?
8. If yes: what would a successful treatment for peanut allergy mean for you and your child if they still had other allergies?

**Wrap up**

1. Are there any other ways in which your or your child’s quality of life is, or has been, impacted by peanut allergy that we have not discussed?

**Thank you for your helpful feedback.**

**We really appreciate the time you have taken to participate in this study.**

**Supplementary Videos**

Interactive results of APPEAL-2 (mp4 file)

- Interactive screen: [https://solaris.devarea.io/appeal2/](https://urldefense.proofpoint.com/v2/url?u=https-3A__solaris.devarea.io_appeal2_&d=DwMFAg&c=UuS_EED7aL1ulQ7-5gKKpA&r=cUxtVVAp9XLaYo7BIsA25ZJQ39RwdlyFMO9ns5pg9WU&m=ORtzv8TsVIsdCNVC7IwVfqXkekZTOKhT4T5O55tWwMw&s=B2QBOM_dGSP_ug6-YrgtFM_oeySHJKe28q6Pjivls-s&e=)

Animated learnings by age group in APPEAL-2 (mp4 files)

- Children: <https://youtu.be/tPqVVXgpYs0>
- Teenager: <https://youtu.be/H81vCuhw8io>
- Adults: <https://youtu.be/8gqxkxJyLDs>

**APPEAL, A**llergy to **P**eanuts Im**P**acting **E**motions **A**nd **L**ife
