# Supplementary material for: APPEAL‐2: A pan‐European qualitative study to explore the burden of peanut‐allergic children, teenagers and their caregivers
Source: Clin Exp Allergy. 2020 Sep 15;50(11):1238–48. doi: 10.1111/cea.13719 (PMC7780296; doi:10.1111/cea.13719)
Supplement: Supplementary file 2 [file CEA-50-1238-s002.zip › index.html]

APPEAL2 Touchscreen


[

](./videos/bg.mp4)

STUDY DESIGN

CHILDREN

TEENAGERS

ADULTS

CAREGIVERS

[

](./videos/toast1.mp4)

1

2

3

4

1

2

3

4

1

2

3

4

1

2

3

4
