# Supplementary figures and images for: APPEAL‐2: A pan‐European qualitative study to explore the burden of peanut‐allergic children, teenagers and their caregivers
Source: Clin Exp Allergy. 2020 Sep 15;50(11):1238–48. doi: 10.1111/cea.13719 (PMC7780296; doi:10.1111/cea.13719)

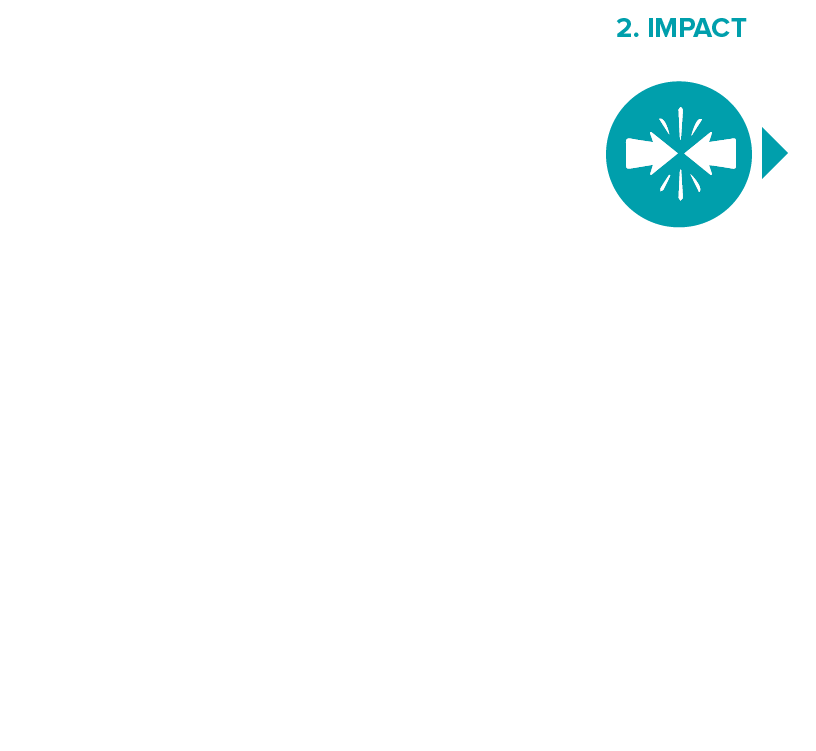

Supplement: Supplementary file 2 [file CEA-50-1238-s002.zip › img/circle2-on.png]

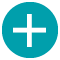

Supplement: Supplementary file 2 [file CEA-50-1238-s002.zip › img/plus.png]

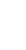

Supplement: Supplementary file 2 [file CEA-50-1238-s002.zip › img/arrow-right.png]

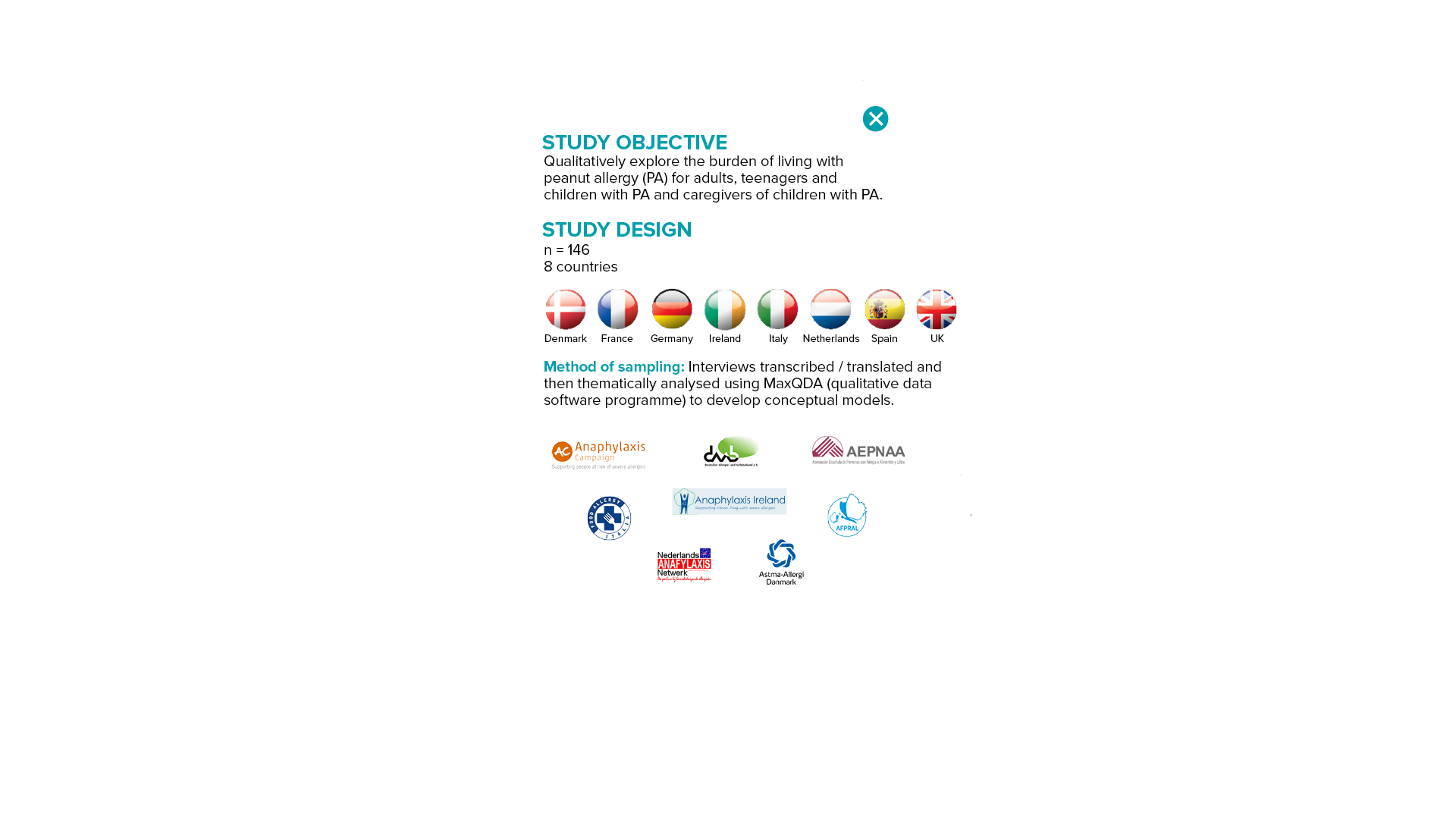

Supplement: Supplementary file 2 [file CEA-50-1238-s002.zip › img/study.png]

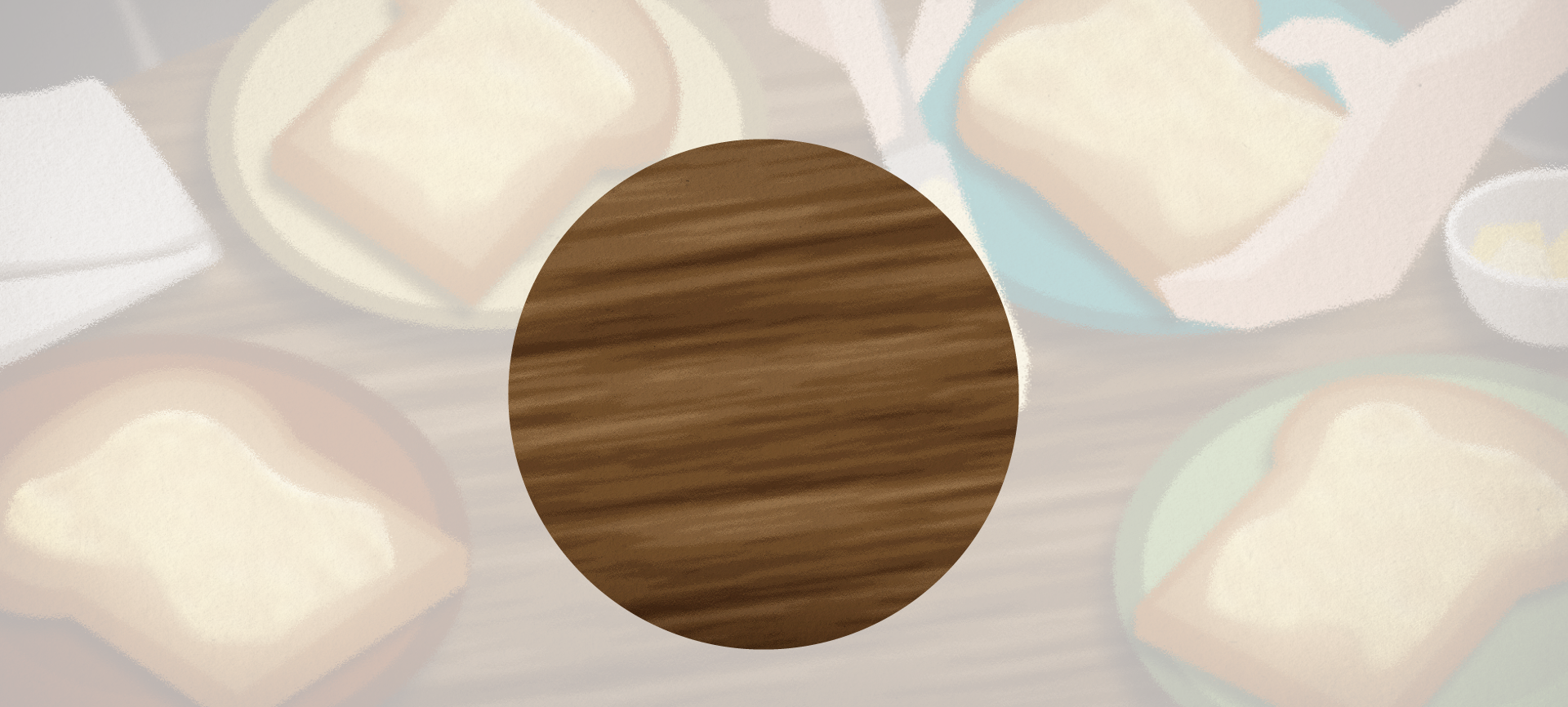

Supplement: Supplementary file 2 [file CEA-50-1238-s002.zip › img/video_bg.png]

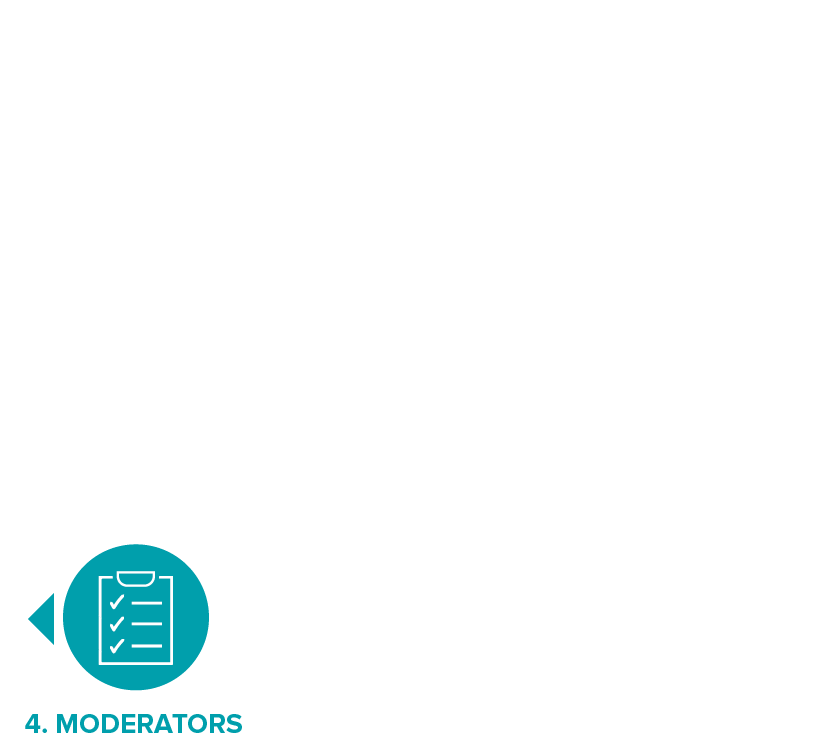

Supplement: Supplementary file 2 [file CEA-50-1238-s002.zip › img/circle4-on.png]

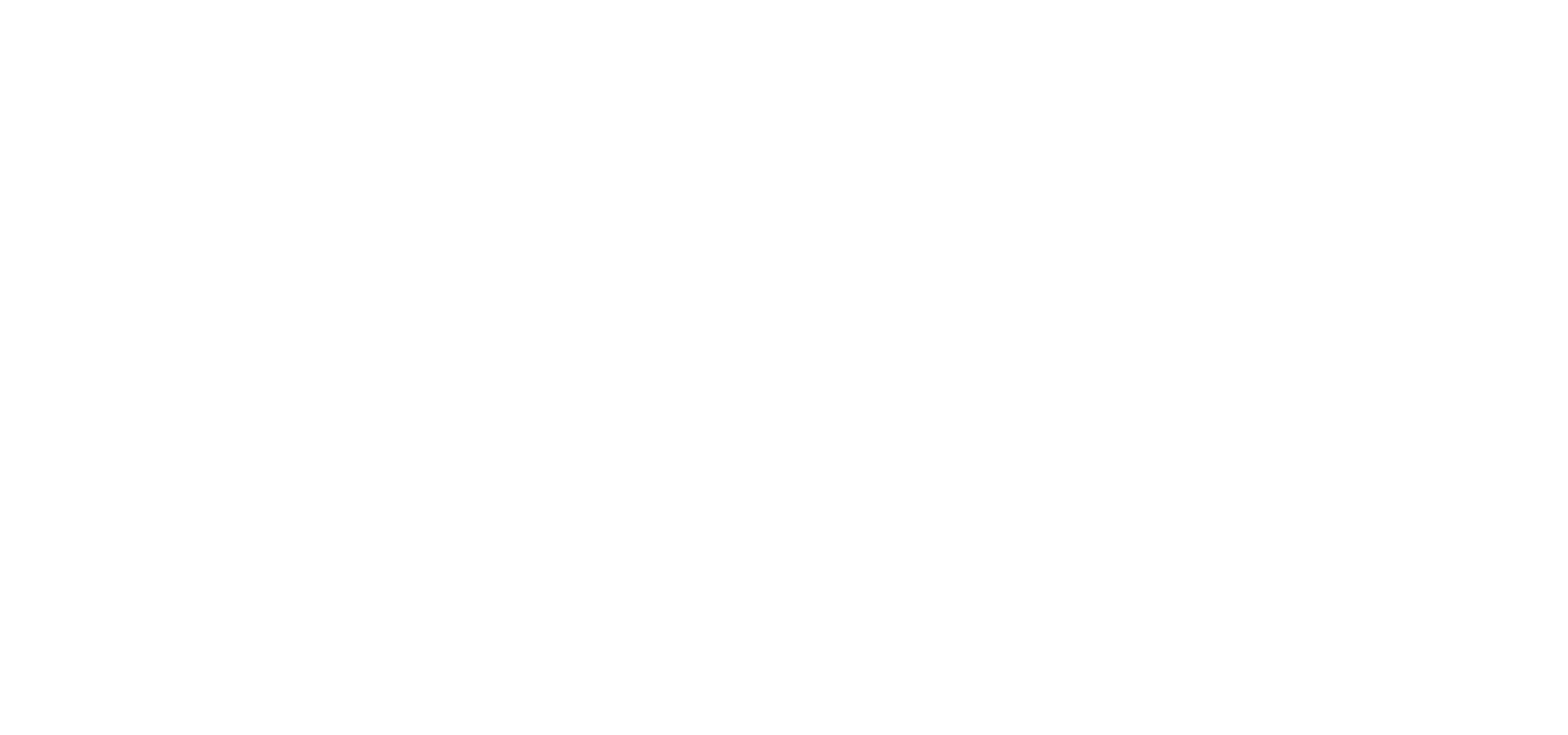

Supplement: Supplementary file 2 [file CEA-50-1238-s002.zip › img/home.png]

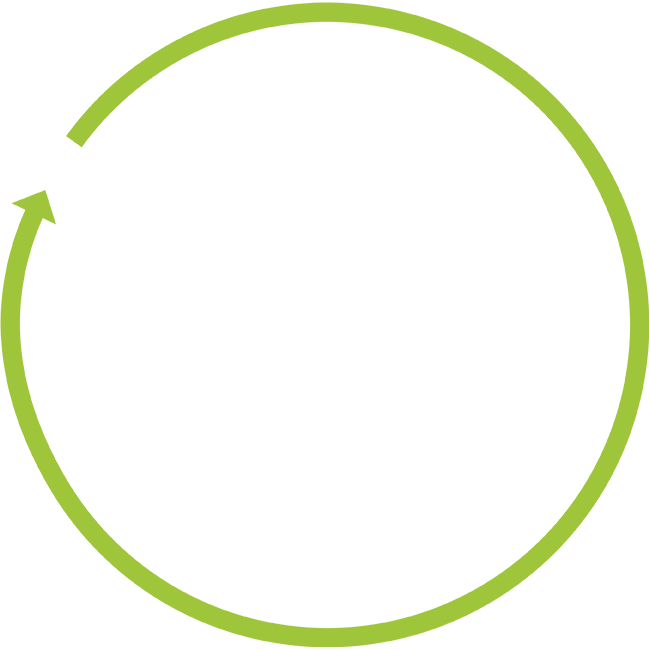

Supplement: Supplementary file 2 [file CEA-50-1238-s002.zip › img/arrow.png]

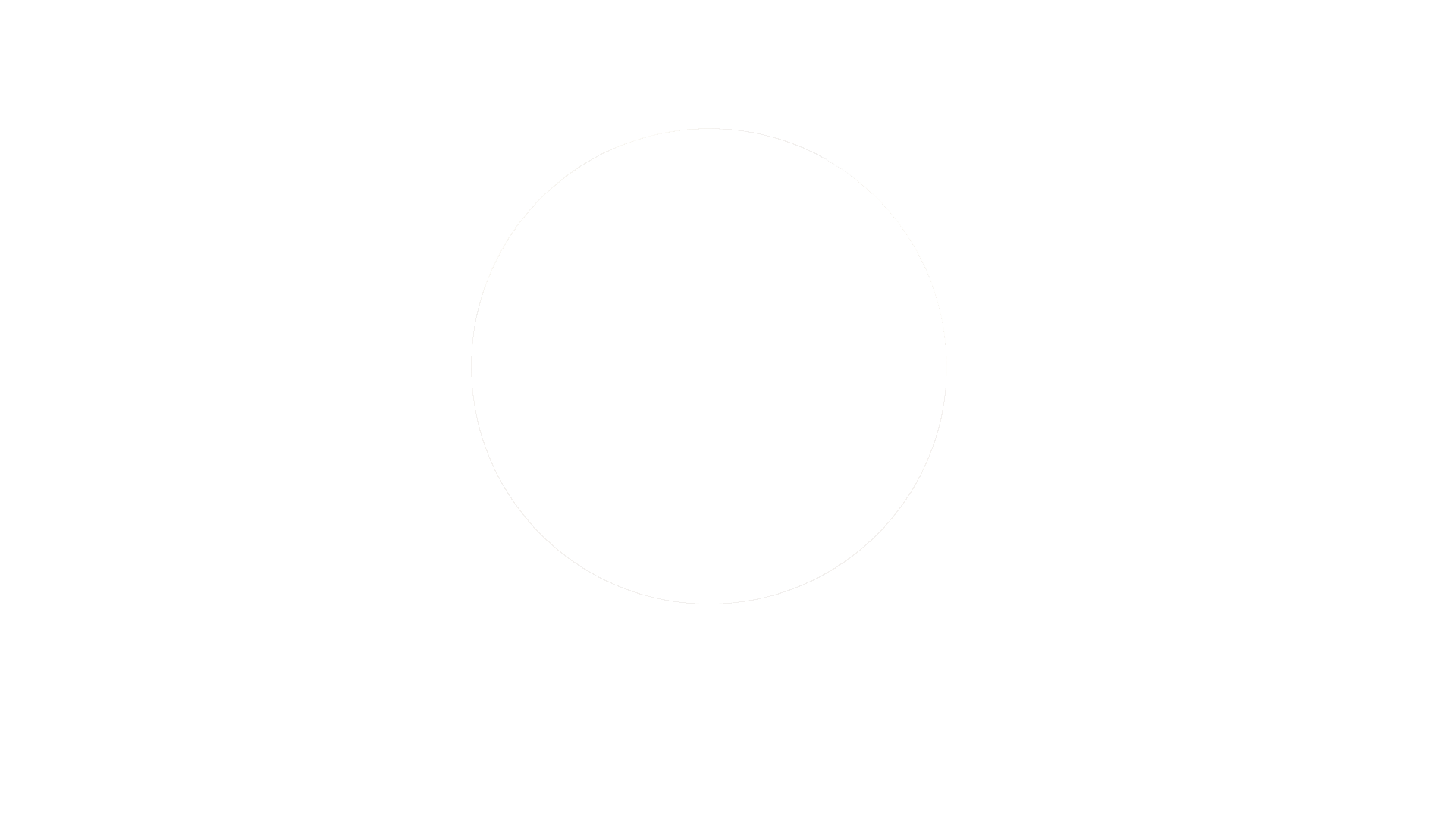

Supplement: Supplementary file 2 [file CEA-50-1238-s002.zip › img/white_mask.png]

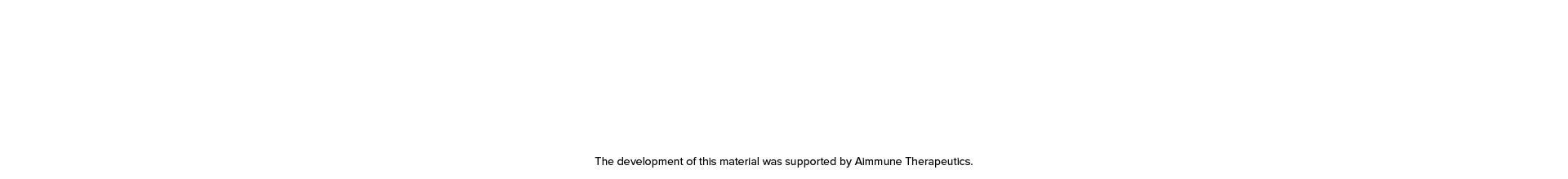

Supplement: Supplementary file 2 [file CEA-50-1238-s002.zip › img/footer-message.png]

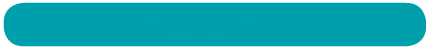

Supplement: Supplementary file 2 [file CEA-50-1238-s002.zip › img/btn-off.png]

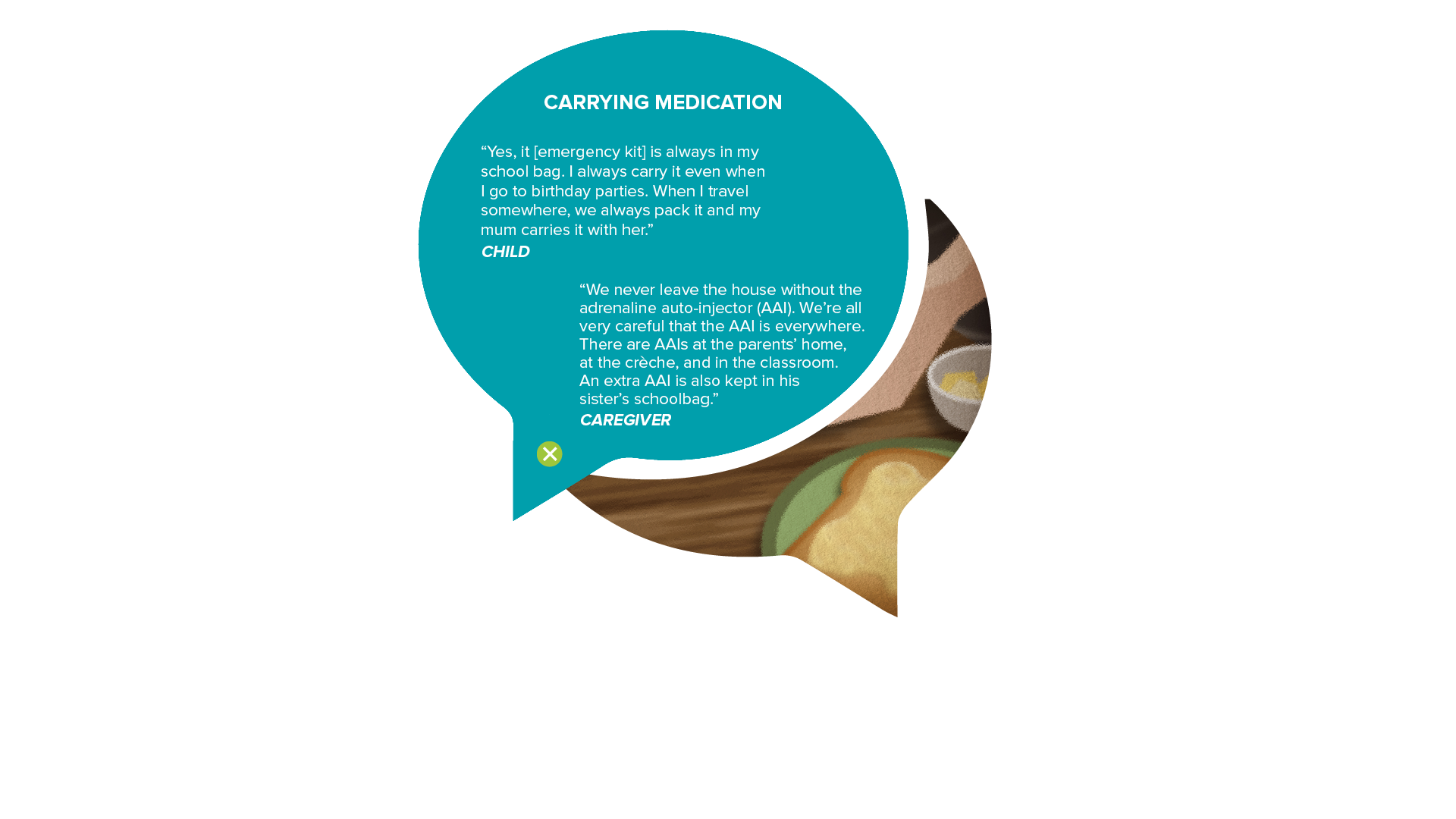

Supplement: Supplementary file 2 [file CEA-50-1238-s002.zip › img/msg4.png]

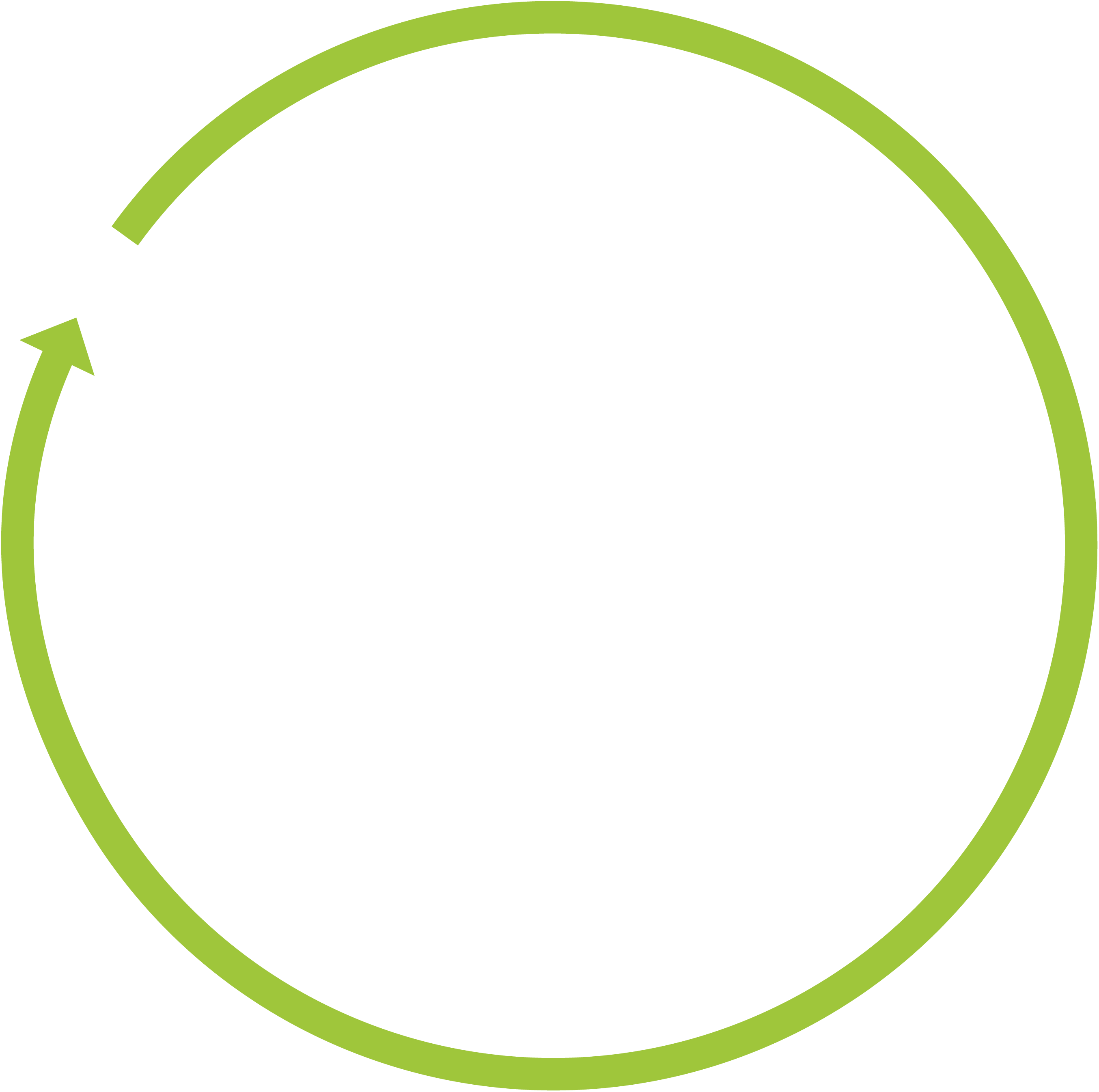

Supplement: Supplementary file 2 [file CEA-50-1238-s002.zip › img/Green Arrow.png]

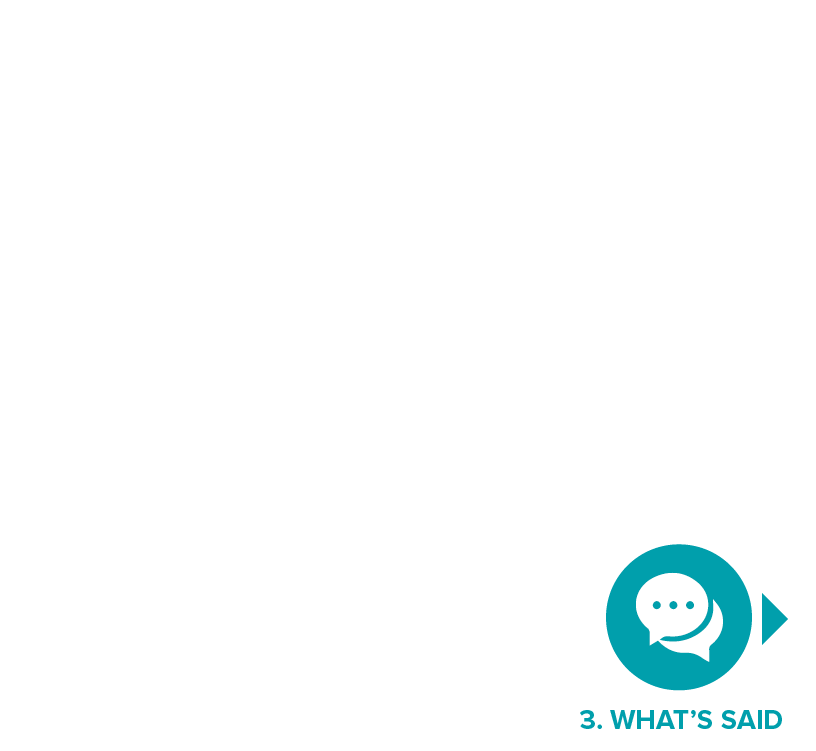

Supplement: Supplementary file 2 [file CEA-50-1238-s002.zip › img/circle3-on.png]

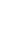

Supplement: Supplementary file 2 [file CEA-50-1238-s002.zip › img/arrow-left.png]

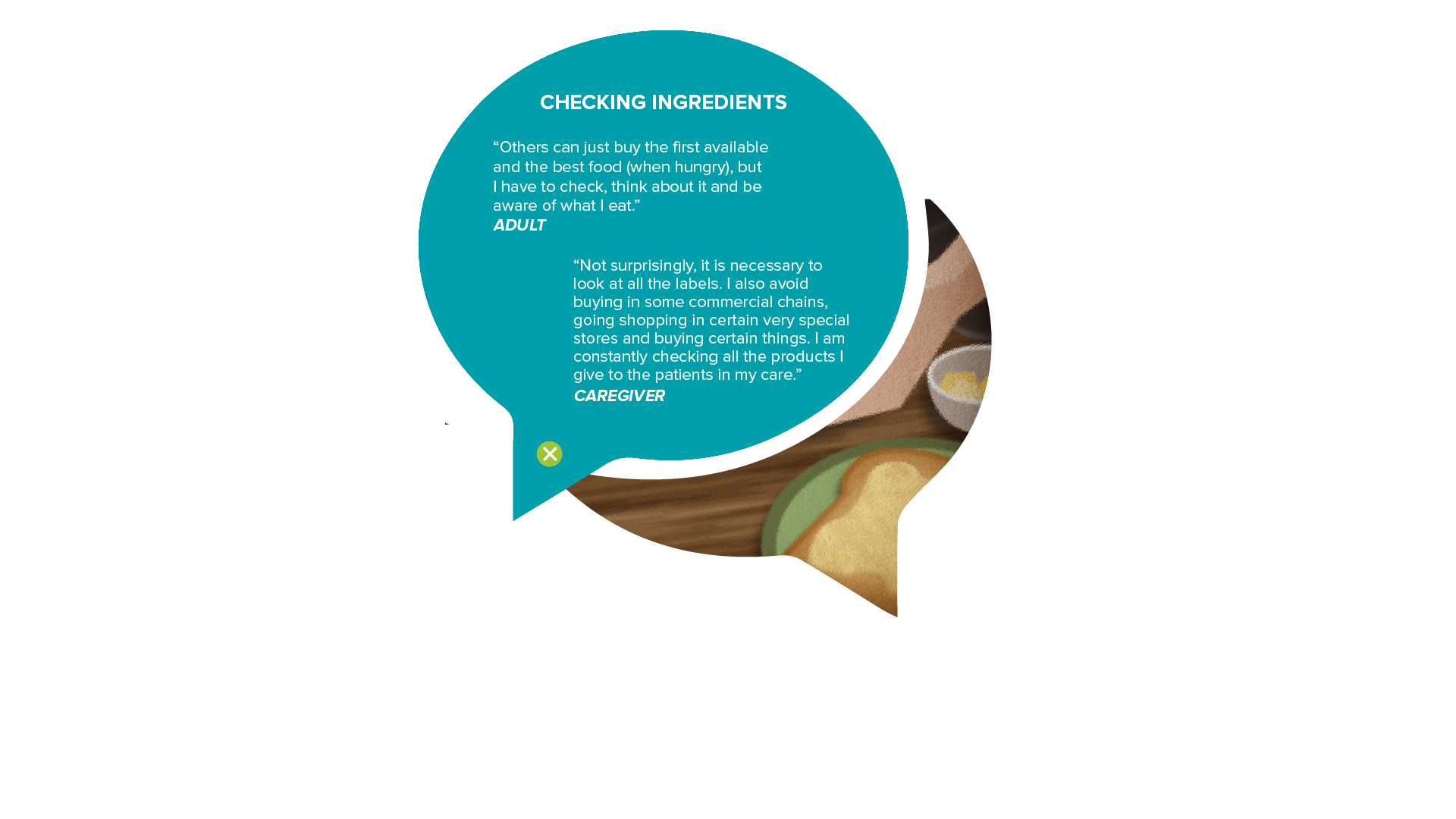

Supplement: Supplementary file 2 [file CEA-50-1238-s002.zip › img/msg3.png]

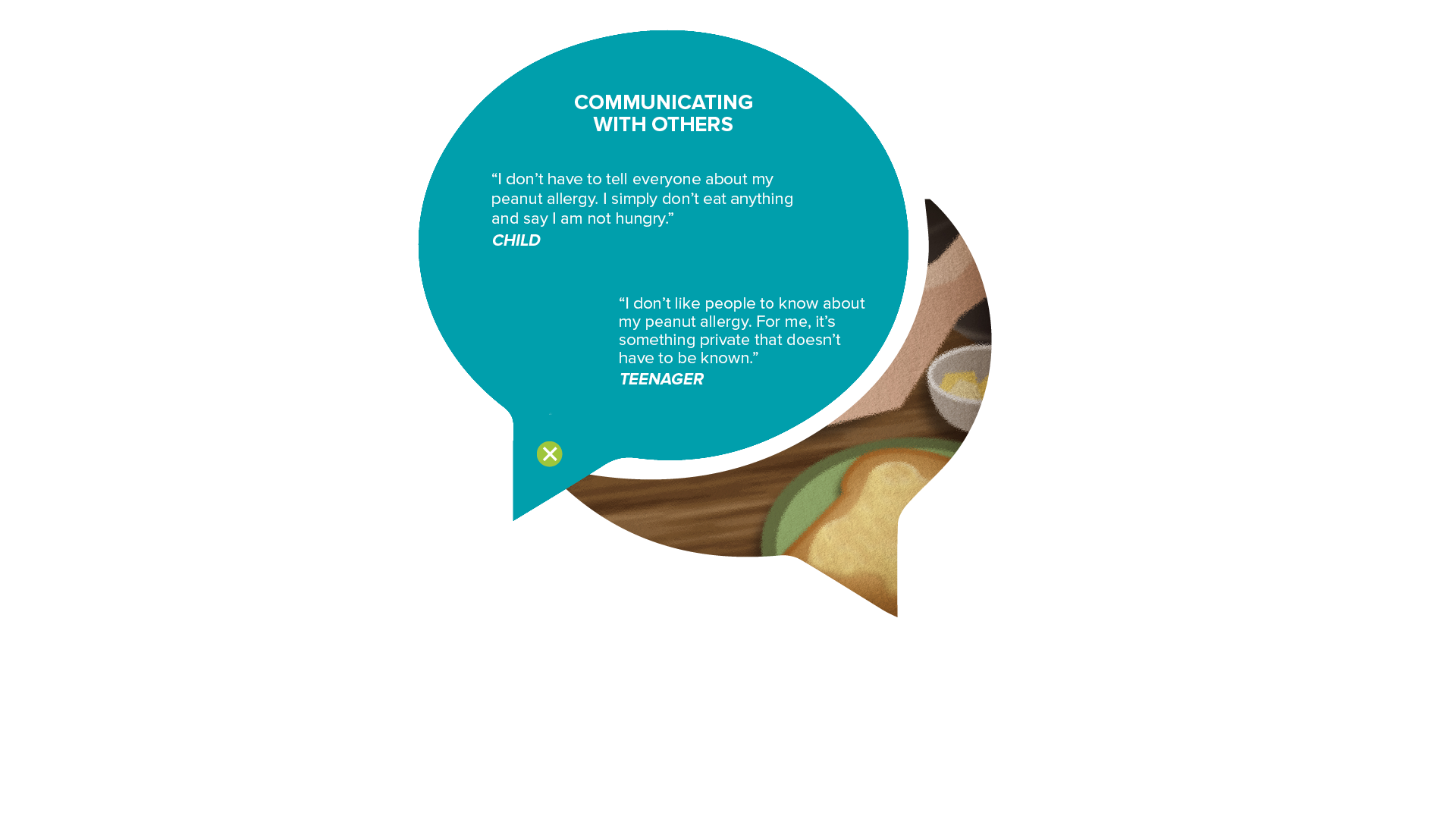

Supplement: Supplementary file 2 [file CEA-50-1238-s002.zip › img/msg2.png]

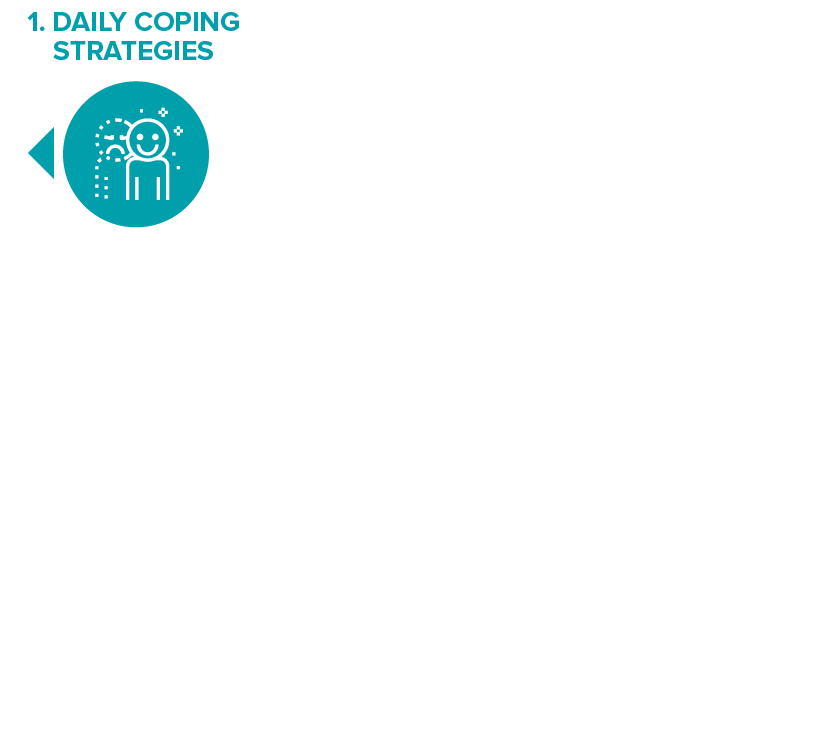

Supplement: Supplementary file 2 [file CEA-50-1238-s002.zip › img/circle1-on.png]

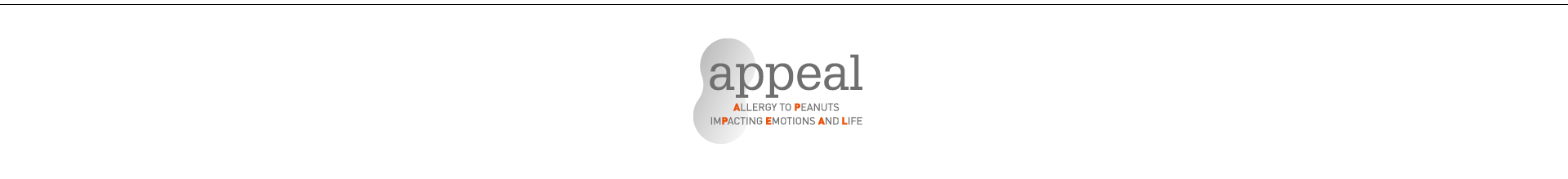

Supplement: Supplementary file 2 [file CEA-50-1238-s002.zip › img/bottom-strip.png]

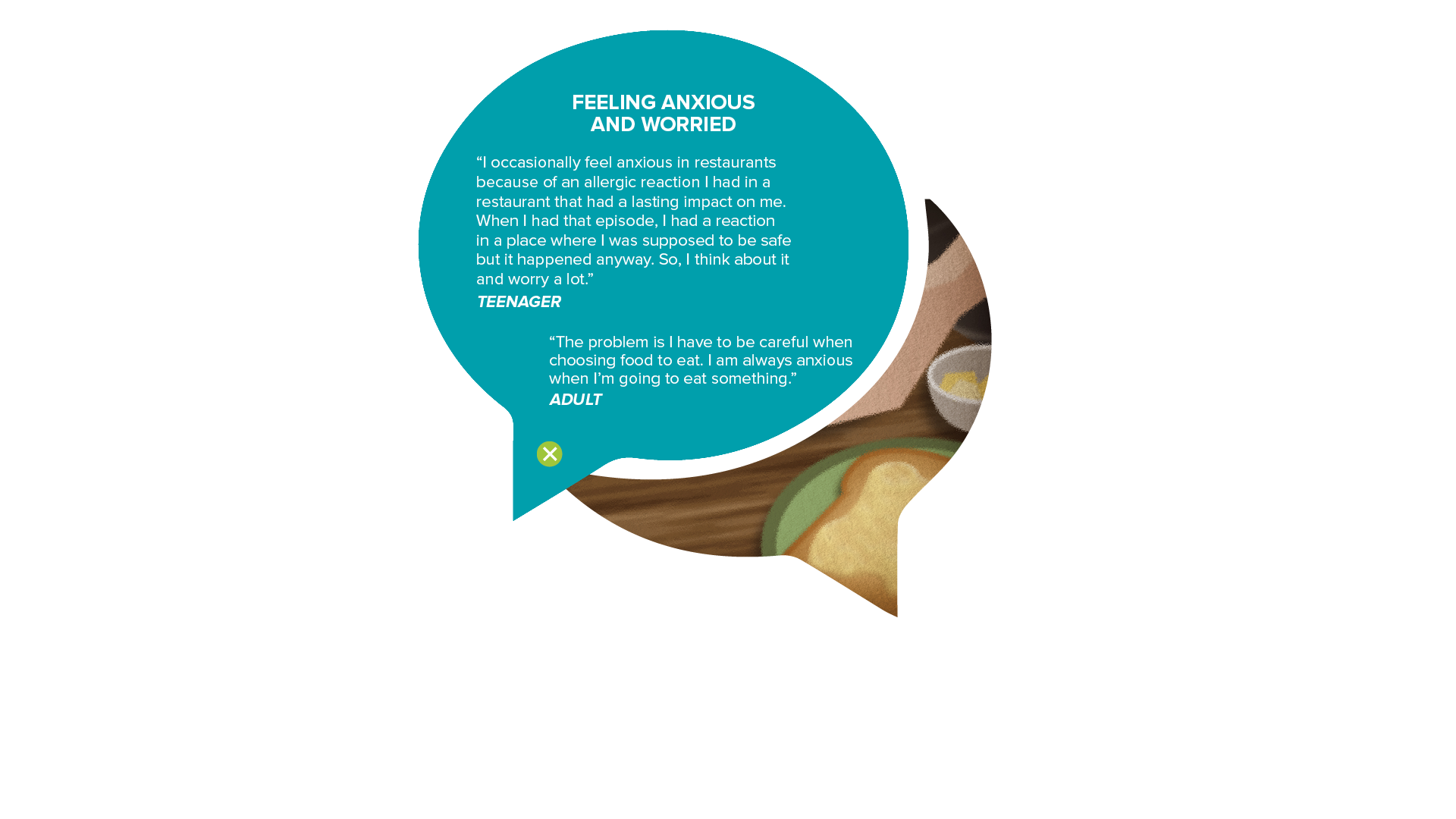

Supplement: Supplementary file 2 [file CEA-50-1238-s002.zip › img/msg1.png]

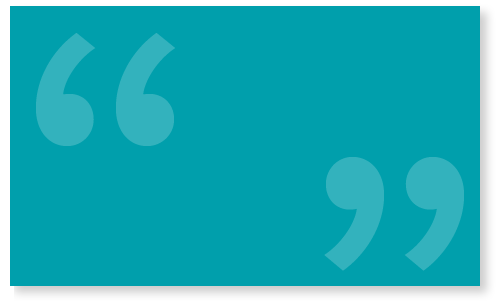

Supplement: Supplementary file 2 [file CEA-50-1238-s002.zip › img/right-card-speech.png]

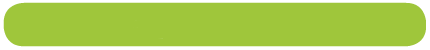

Supplement: Supplementary file 2 [file CEA-50-1238-s002.zip › img/btn-on.png]

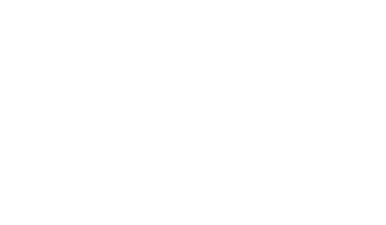

Supplement: Supplementary file 2 [file CEA-50-1238-s002.zip › img/card-content/teen/teen4.png]

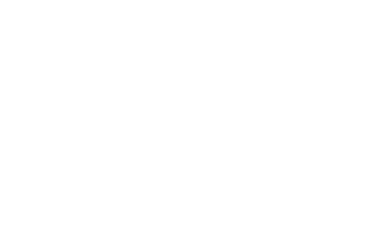

Supplement: Supplementary file 2 [file CEA-50-1238-s002.zip › img/card-content/teen/teen1-1.png]

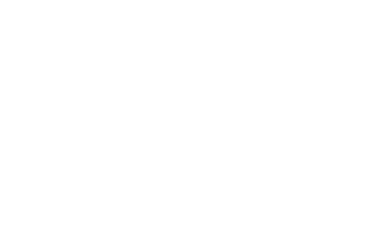

Supplement: Supplementary file 2 [file CEA-50-1238-s002.zip › img/card-content/teen/teen3-2.png]

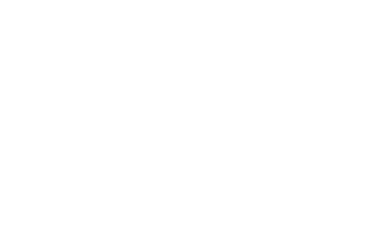

Supplement: Supplementary file 2 [file CEA-50-1238-s002.zip › img/card-content/teen/teen1-2.png]

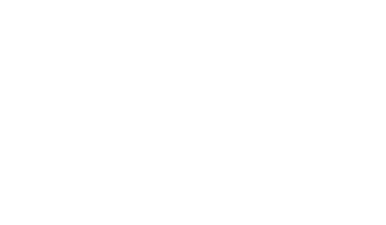

Supplement: Supplementary file 2 [file CEA-50-1238-s002.zip › img/card-content/teen/teen1-3.png]

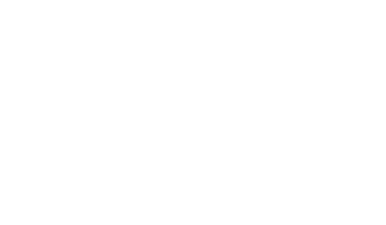

Supplement: Supplementary file 2 [file CEA-50-1238-s002.zip › img/card-content/teen/teen3-1.png]

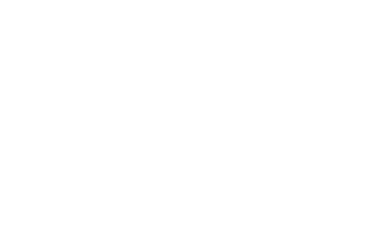

Supplement: Supplementary file 2 [file CEA-50-1238-s002.zip › img/card-content/teen/teen2-1.png]

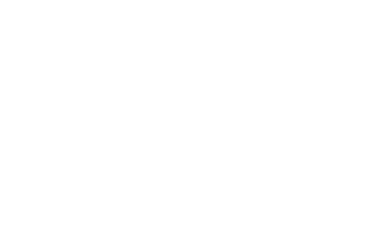

Supplement: Supplementary file 2 [file CEA-50-1238-s002.zip › img/card-content/teen/teen2-3.png]

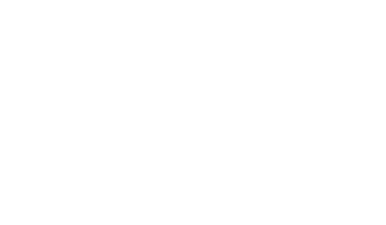

Supplement: Supplementary file 2 [file CEA-50-1238-s002.zip › img/card-content/teen/teen2-2.png]

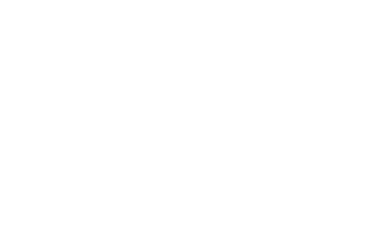

Supplement: Supplementary file 2 [file CEA-50-1238-s002.zip › img/card-content/adults/adult2-3.png]

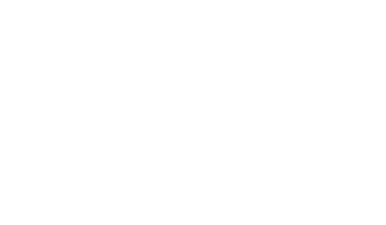

Supplement: Supplementary file 2 [file CEA-50-1238-s002.zip › img/card-content/adults/adult2-2.png]

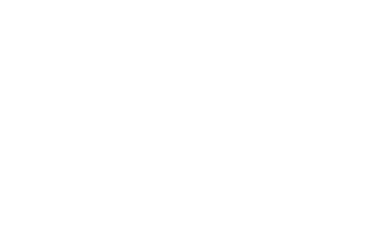

Supplement: Supplementary file 2 [file CEA-50-1238-s002.zip › img/card-content/adults/adult2-1.png]

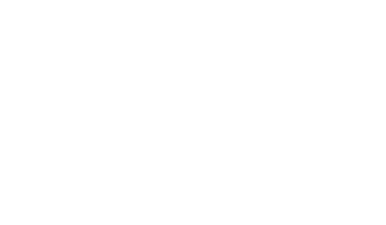

Supplement: Supplementary file 2 [file CEA-50-1238-s002.zip › img/card-content/adults/adult1-2.png]

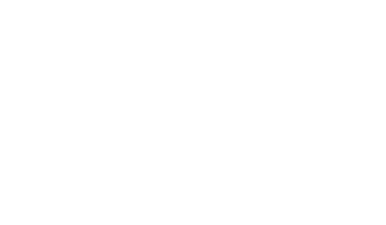

Supplement: Supplementary file 2 [file CEA-50-1238-s002.zip › img/card-content/adults/adult3-1.png]

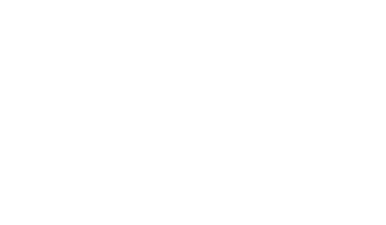

Supplement: Supplementary file 2 [file CEA-50-1238-s002.zip › img/card-content/adults/adult1-3.png]

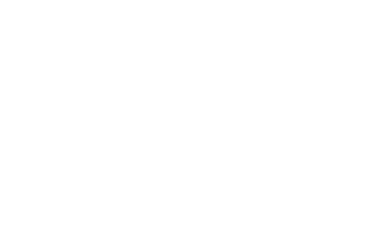

Supplement: Supplementary file 2 [file CEA-50-1238-s002.zip › img/card-content/adults/adult1-1.png]

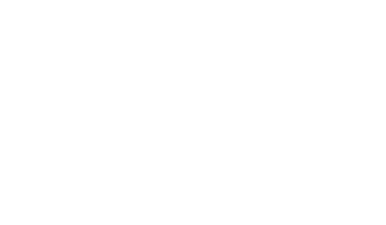

Supplement: Supplementary file 2 [file CEA-50-1238-s002.zip › img/card-content/adults/adult4.png]

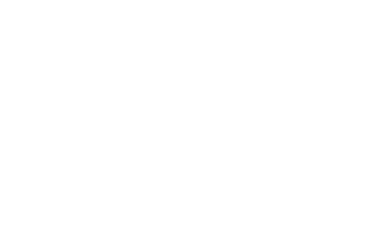

Supplement: Supplementary file 2 [file CEA-50-1238-s002.zip › img/card-content/adults/adult3-2.png]

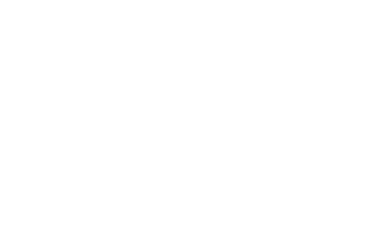

Supplement: Supplementary file 2 [file CEA-50-1238-s002.zip › img/card-content/caregivers/care2-3.png]

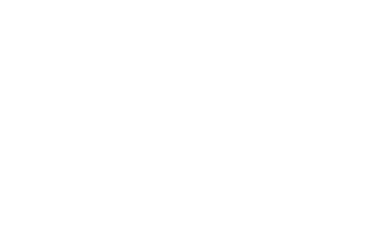

Supplement: Supplementary file 2 [file CEA-50-1238-s002.zip › img/card-content/caregivers/care2-2.png]

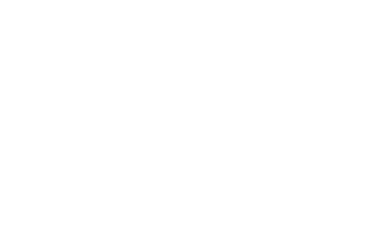

Supplement: Supplementary file 2 [file CEA-50-1238-s002.zip › img/card-content/caregivers/care4.png]

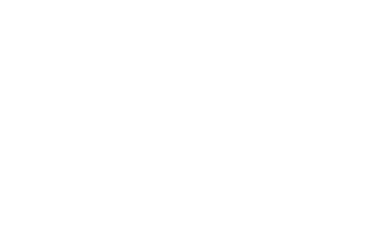

Supplement: Supplementary file 2 [file CEA-50-1238-s002.zip › img/card-content/caregivers/care2-1.png]

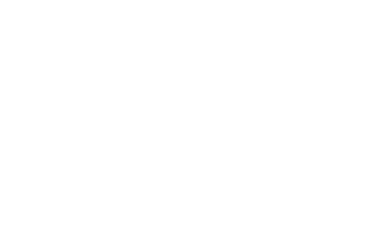

Supplement: Supplementary file 2 [file CEA-50-1238-s002.zip › img/card-content/caregivers/care2-4.png]

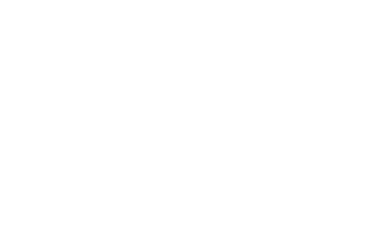

Supplement: Supplementary file 2 [file CEA-50-1238-s002.zip › img/card-content/caregivers/care1-2.png]

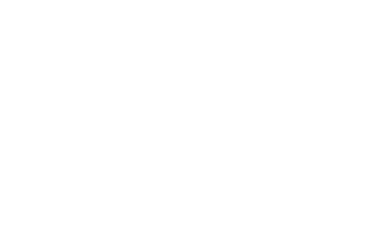

Supplement: Supplementary file 2 [file CEA-50-1238-s002.zip › img/card-content/caregivers/care3-1.png]

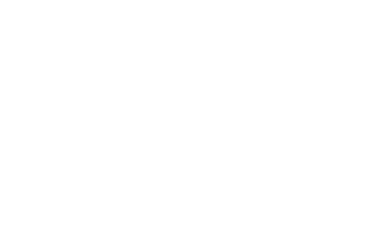

Supplement: Supplementary file 2 [file CEA-50-1238-s002.zip › img/card-content/caregivers/care1-3.png]

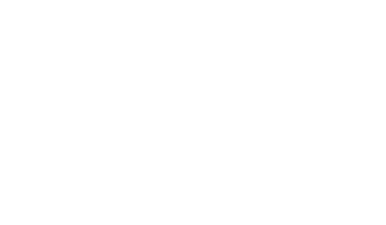

Supplement: Supplementary file 2 [file CEA-50-1238-s002.zip › img/card-content/caregivers/care1-1.png]

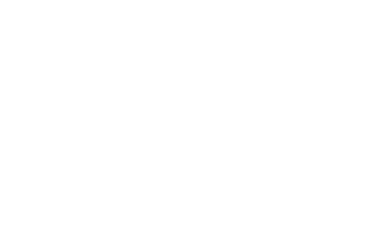

Supplement: Supplementary file 2 [file CEA-50-1238-s002.zip › img/card-content/caregivers/care3-2.png]

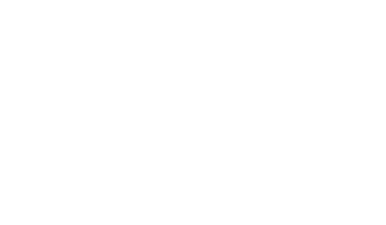

Supplement: Supplementary file 2 [file CEA-50-1238-s002.zip › img/card-content/children/child1-1.png]

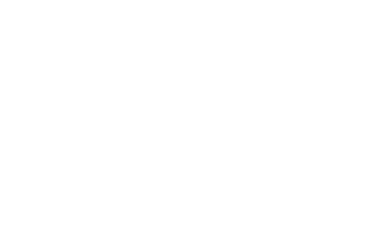

Supplement: Supplementary file 2 [file CEA-50-1238-s002.zip › img/card-content/children/child3-2.png]

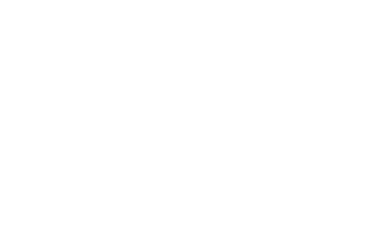

Supplement: Supplementary file 2 [file CEA-50-1238-s002.zip › img/card-content/children/child1-2.png]

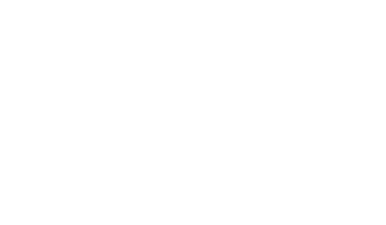

Supplement: Supplementary file 2 [file CEA-50-1238-s002.zip › img/card-content/children/child1-3.png]

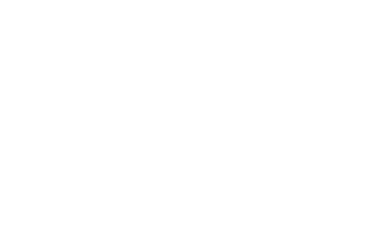

Supplement: Supplementary file 2 [file CEA-50-1238-s002.zip › img/card-content/children/child3-1.png]

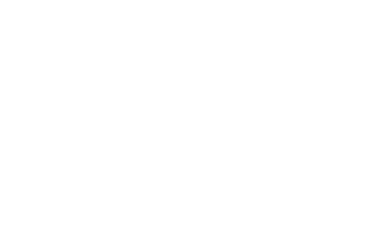

Supplement: Supplementary file 2 [file CEA-50-1238-s002.zip › img/card-content/children/child2-1.png]

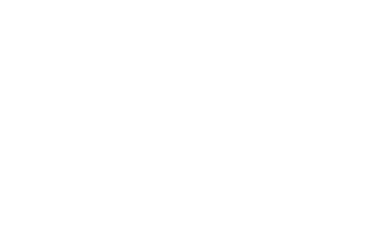

Supplement: Supplementary file 2 [file CEA-50-1238-s002.zip › img/card-content/children/child2-3.png]

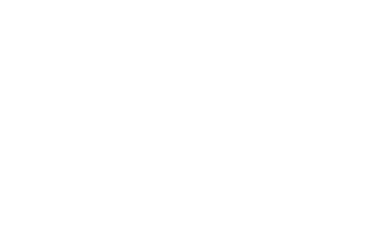

Supplement: Supplementary file 2 [file CEA-50-1238-s002.zip › img/card-content/children/child2-2.png]

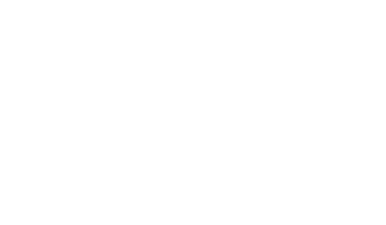

Supplement: Supplementary file 2 [file CEA-50-1238-s002.zip › img/card-content/children/child4.png]

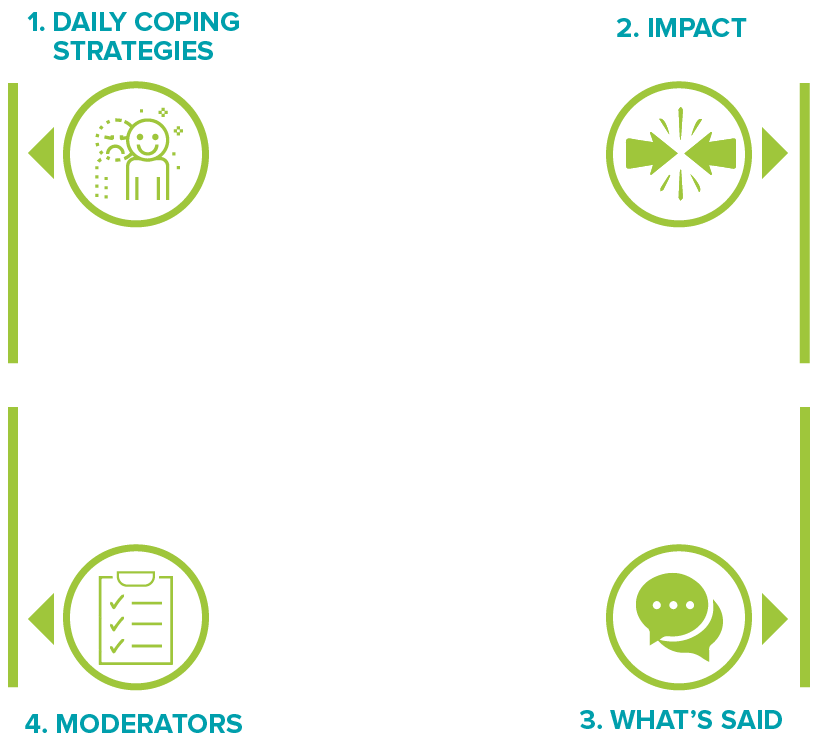

Supplement: Supplementary file 2 [file CEA-50-1238-s002.zip › img/circles-off.png]

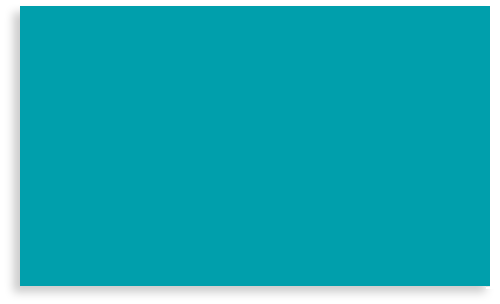

Supplement: Supplementary file 2 [file CEA-50-1238-s002.zip › img/left-card.png]

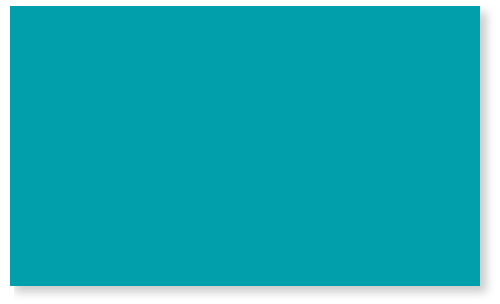

Supplement: Supplementary file 2 [file CEA-50-1238-s002.zip › img/right-card.png]
